# IL‐33 Facilitates Fibro‐Adipogenic Progenitors to Establish the Pro‐Regenerative Niche after Muscle Injury

**DOI:** 10.1002/advs.202405299

**Published:** 2024-07-22

**Authors:** Congcong Zhang, Guoqi Li, Fan Zhang, Yanhong Zhang, Shiyao Hong, Shijuan Gao, Yan Liu, Jie Du, Yulin Li

**Affiliations:** ^1^ Beijing Anzhen Hospital Capital Medical University Beijing Institute of Heart Lung and Blood Vessel Diseases Beijing 100029 China; ^2^ Key Laboratory of Remodeling‐related Cardiovascular Diseases Ministry of Education Beijing 100029 China

**Keywords:** fibro‐adipogenic progenitors, IL‐33, inflammation, muscle regeneration

## Abstract

During the process of muscle regeneration post‐injury in adults, muscle stem cells (MuSCs) function is facilitated by neighboring cells within the pro‐regenerative niche. However, the precise mechanism triggering the initiation of signaling in the pro‐regenerative niche remains unknown. Using single‐cell RNA sequencing, 14 different muscle cells are comprehensively mapped during the initial stage following injury. Among these, macrophages and fibro‐adipogenic progenitor cells (FAPs) exhibit the most pronounced intercellular communication with other cells. In the FAP subclusters, the study identifies an activated FAP phenotype that secretes chemokines, such as CXCL1, CXCL5, CCL2, and CCL7, to recruit macrophages after injury. *Il1rl1*, encoding the protein of the interleukin‐33 (IL‐33) receptor, is identified as a highly expressed signature surface marker of the FAP phenotype. Following muscle injury, autocrine IL‐33, an alarmin, has been observed to activate quiescent FAPs toward this inflammatory phenotype through the IL1RL1‐MAPK/NF‐κB signaling pathway. *Il1rl1* deficiency results in decreased chemokine expression and recruitment of macrophages, accompanied by impaired muscle regeneration. These findings elucidate a novel mechanism involving the IL‐33/IL1RL1 signaling pathway in promoting the activation of FAPs and facilitating muscle regeneration, which can aid the development of therapeutic strategies for muscle‐related disorders and injuries.

## Graphical abstract

Single‐cell RNA sequencing of injured muscles reveals that FAPs differentiate into an activated phenotype to chemoattract leukocytes to promote the establishment of a regenerative niche following injury. IL‐33 promotes the FAPs activation and leukocytes recruitment by IL1RL1‐MAPKp38‐NF‐κB signaling pathway. Loss of IL1RL1 impairs muscle regeneration and increases matrix deposition after injury.

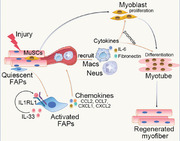

## 1 Introduction

Skeletal muscle is the most abundant tissue in the human body and stores nearly 70% of the body's protein, serving as a vital nutrition source. Adult muscles exhibit remarkable regenerative ability to maintain muscle mass and function in response to several types of injuries (such as strains, wounds, and lacerations). Tissue‐resident muscle stem cells (MuSCs), which are located in a specialized niche beneath the sarcolemma and basal lamina of myofibers, regulate muscle regeneration.^[^ ^1^ ^]^ MuSCs are mitotically quiescent and undergo activation, proliferation, and differentiation to form new myofibers after injury.^[^ ^2^, ^3^ ^]^ Insufficient muscle regeneration following injury results in a loss of muscle strength and mass.^[^ ^4^, ^5^ ^]^ Therefore, understanding the molecular mechanisms underlying muscle regeneration after injury is essential for maintaining muscle function.

In addition to the autonomous transcriptional regulation of MuSCs, their activity is also regulated by niche cells within the muscles.^[^ ^3^, ^5^ ^]^ After muscle injury, circulating neutrophils and Ly6C^hi^ pro‐inflammatory mono/macrophages are recruited to the muscle by chemokines, such as CCL2, CCL3, and CXCL16),^[^ ^6^, ^7^, ^8^ ^]^ to phagocytose necrotic myofibers. Subsequently, Ly6C^hi^ pro‐inflammatory macrophages are converted into Ly6C^low^ anti‐inflammatory macrophages to promote the proliferation and differentiation of MuSCs.^[^ ^9^, ^10^ ^]^ Pharmacologic or genetic depletion of macrophages impairs muscle regeneration.^[^ ^9^, ^11^ ^]^ In one of our recent studies, we demonstrated that the activation of the complement system in injured myofibers and complement C3a increased the expression of the chemokine CCL5 in macrophages, further amplifying the inflammatory response.^[^ ^12^ ^]^ Treg and CD8^+^ T cells also infiltrate injured muscles to interact with macrophages and modulate the inflammatory environment.^[^ ^13^, ^14^ ^]^ However, the initiation of the pro‐regenerative immune response remains incompletely understood.

In addition to immune cells, fibro‐adipogenic progenitor cells (FAPs) are rapidly expanding niche cell types that augment MuSC activities during response to injury.^[^ ^15^, ^16^ ^]^ FAPs are activated and enter the proliferative phase simultaneously, as MuSCs, and regulated through the secretion of pro‐myogenic cytokines, such as interleukin (IL)−6, follistatin, and WISP1, and extracellular matrix (ECM).^[^ ^16^, ^17^, ^18^ ^]^ Altered FAP differentiation into the fibrogenic lineage during regeneration perturbs ECM remodeling and impairs myogenesis.^[^ ^19^, ^20^ ^]^ FAPs secrete IL‐33 cytokines, which increase Treg cell proliferation and promote aged muscle repair.^[^ ^21^ ^]^ Additionally, FAP‐secreted BMP1 and MMP14 have been shown to increase TGF‐β production by macrophages in fibrotic Duchenne muscular dystrophy muscles.^[^ ^22^ ^]^ These observations demonstrate the potential of FAPs to modulate immune responses, highlighting the need for a comprehensive understanding of their precise role in pro‐regenerative niches.

In this study, using single‐cell RNA‐sequencing (scRNA‐seq), we comprehensively mapped 14 different muscle cells during the early stage of muscle injury. We found that macrophages and FAPs exhibited the most pronounced intercellular communication with other cells within the injured muscle microenvironment. In the FAP cluster, we described an activated FAP (aFAP) subset that secretes chemokines via autocrine IL‐33‐IL1RL1‐MAPKp38/NF‐κB signaling pathway to recruit macrophages and neutrophils following injury. *Il1rl1* deficiency resulted in decreased chemokine expression and recruitment of inflammatory cells, accompanied by impaired MuSC proliferation and compromised muscle regeneration. In summary, we identified an FAP‐derived initial signal that establishes the muscle regenerative niche after injury.

## 2 Results

### 2.1 Single‐Cell Characterization of Skeletal Muscle Cells after Injury

**Figure 1:**
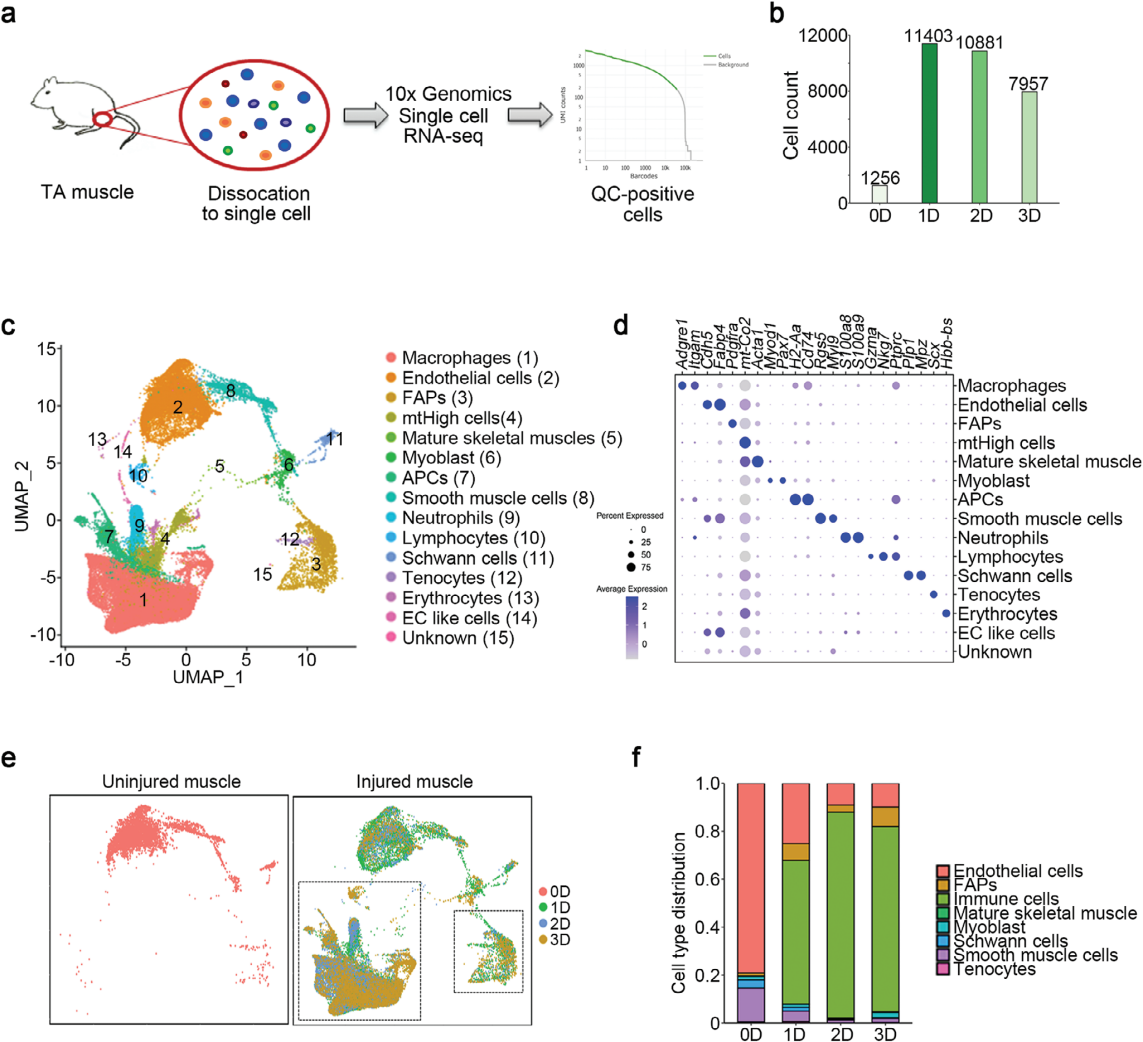
Single‐cell characterization of skeletal muscle cells after injury. a) Cells isolated from uninjured (0D) and cardiotoxin (CTX)‐injured (1D, 2D, 3D) muscles were used for library preparation of single‐cell RNA‐sequences using the 10X Genomic platform. Low‐quality cells and doublets were removed, retaining only quality control (QC)‐positive cells for further analysis. b) The number of QC‐positive cells identified at each time point. c) UMAP representation of the integrated sample dataset. Fifteen cell clusters were identified by the top differentiation expressed marker genes. d) Dot plot showing the expression frequency and average expression level of differentiation expressed marker genes in each cluster. e) The UMAP of cells from uninjured and injured muscles. Cells were colored by time point. f) Fractional distribution of cells from each time point.

The cellular diversity of the skeletal muscles after injury was investigated using a well‐established mouse model of acute muscle injury induced by cardiotoxin (CTX) injection into the tibialis anterior (TA) muscle. Single‐cell suspensions from TA muscles were prepared enzymatically on days 0, 1, 2, and 3 post‐injury. The cells were subjected to scRNA‐seq using the 10x Genomics platform, followed by quality control filtration (**Figure** **1**a,b; Figure S1a, Supporting Information). The Cell Ranger algorithm was applied to identify homogeneous and robust groups of cells from the scRNA‐seq data. All cells were classified into 14 groups based on the expression levels of the most differentially expressed genes (DEGs) (Figure 1c). Known marker genes belonging to distinct cell populations were used to identify muscle cell clusters, including macrophages: *Itgam* (encoding CD11b) and *Adgre1* (encoding F4/80); endothelial cells: *Cdh5* and *Fapb4*; FAPs: *Pdgfra* and *Ly6a* (encoding Sca‐1); MuSCs/myoblasts, *Pax7* or *Itga7*; APCs, *Cd74* and *H2‐Aa*; neutrophils, *S100a8* and *S100a9*; lymphocytes, *Gzma* and *Nkg7*; and Schwann cells: *Mpz* and *Plp* (Figure 1d). We then compared the uninjured (day 0) and injured (days 1, 2, and 3) muscle cell clusters and found a significant increase in the number of immune cells and FAPs (Figure 1e,f). Altogether, these findings provide comprehensive single‐cell gene expression profiles of injured muscles that can identify all distinct MuSC niche cells.

### 2.2 aFAPs Secrete Chemokines to Recruit Macrophages and Neutrophils after Injury

**Figure 2:**
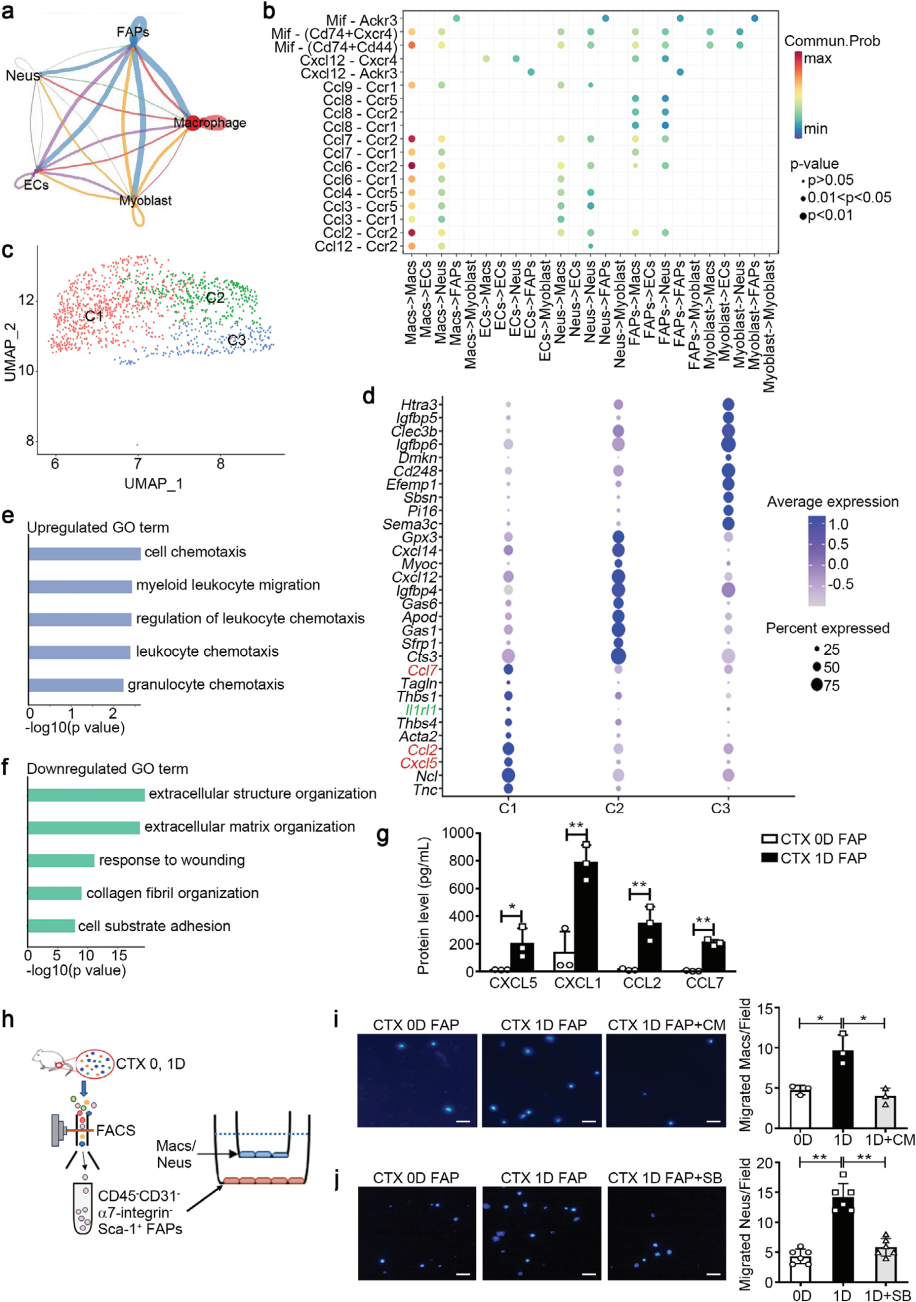
Activated FAPs secrete chemokines to recruit macrophages after injury. a) CellChat DB was used for cell‐cell communication analysis in the five main cell types of the injured muscle. The thickness of the connecting line represents the number of interactions between cells. b) Dot plot representations of the chemokine and chemokine receptor interaction pairs between different muscle cell types on day 1 after injury. c) The UMAP plot showed that FAPs were further divided into three clusters (C1, C2, and C3) based on differentially expressed genes. d) Dot plot showing the expression frequency and average expression levels of the top 10 differentially expressed genes in the three clusters. e,f) GO function analysis of upregulated (e) and downregulated genes (f) in the C1 cluster compared with the other two clusters. g) CD45^−^CD31^−^α7‐integrin^−^Sca‐1^+^ FAPs were sorted from muscles on days 0 and 1 after the CTX injury. The protein levels of CXCL5, CXCL1, CCL2, and CCL7 in the culture supernatants of FAPs were measured at 12 h using a cytokine multiplex assay (n = 3 samples per time point). h) After sorting from muscles, 3 ×10^4^ FAPs were seeded in the lower chambers of the Transwell system for 12 h, subsequently, 1 × 10^4^ macrophages (Macs) or neutrophils (Neus) per well were seeded into the upper chamber. i,j) Migrated macrophages (i) or neutrophils (j) per image were quantified using DAPI staining at 12 h after co‐culture. Scale bar, 100 µm (n = 3–6 samples per group, ten images per sample). Data are represented as mean ± s.e.m. *p < 0.05, **p < 0.01 by unpaired Student's *t*‐test. CM, Cenicriviroc Mesylate; SB, SB225002.

To identify the potential interactions between activated cells after injury, we analyzed the expression patterns of corresponding receptors and ligands in the five main cell types. Interaction pairs were mainly distributed between macrophages and FAPs at an early stage (**Figure** **2**a). For instance, on day 1 post‐injury, the chemokine and chemokine receptor interactions existed mainly between “Macs to Macs” and “FAPs to Macs” (Figure 2b; Figure S1b,c, Supporting Information). Considering that macrophages are known to be a source of chemokines, we further analyzed FAP functions on day 1. Subcluster analysis of FAPs revealed three subclusters on this day (Figure 2c). As shown in Figure 2d, the top 10 DEGs in the C1 cluster (47.8% of FAPs) included the chemokine genes *Cxcl5*, *Ccl2*, and *Ccl7* (Figure 2d). There were 44 upregulated and 71 downregulated DEGs in the C1 cluster of FAPs (*p*‐value < 0.05, Figure S2a, Table S1, Supporting Information). Subsequently, Gene Ontology (GO) analysis of the DEGs also indicated that the upregulated GO terms included cell chemotaxis and myeloid leukocyte migration pathways, while the downregulated GO terms included extracellular structure organization and ECM organization (Figure 2e,f).

Furthermore, we verified the expression levels of DEGs in “cell chemotaxis” terms at different time points after muscle injury. The mRNA levels of the chemokine genes, such as CXCL5, CXCL1, CCL2, MIF, and CCL7, in the muscle increased significantly and peaked on day 1 post‐injury (Figure S2b–f, Supporting Information). The protein levels of chemokines, including CXCL5, CXCL1, CCL2, and CCL7, in injured muscles and serum increased significantly on day 1 post‐injury (Figure S2g,h, Supporting Information). Thereafter, we isolated CD45^−^CD31^−^α7‐integrin^−^Sca‐1^+^ FAPs from muscles on day 0 and day 1 post‐injury (Figure S2i, Supporting Information) and discovered that FAPs from day‐1 muscles had higher mRNA and protein levels of these chemokines compared to those from day‐0 muscles (Figure 2g; Figure S2j, Supporting Information). In addition to these chemokines, the levels of other cytokines such as IL‐6, IL‐18, G‐CSF, and LIF were also increased in the culture medium of FAPs sorted from injured day‐1 muscles (Table S2, Supporting Information). These data indicated the presence of an aFAP phenotype in the injured muscle.

The common receptor of CXCL5 and CXCL1 is CXCR2, and the common receptors of CCL2 and CCL7 are CCR2 and CCR5.^[^ ^23^ ^]^ In injured muscles, there were more CD11b^+^CXCR2^+^ and CD11b^+^CCR2^+^ cells compared to normal muscles (Figure S3a,b, Supporting Information). CD11b^+^CXCR2^+^ cells were F4/80^−^Ly6G^+^ neutrophils and F4/80^+^Ly6G^+^ macrophages, while CD11b^+^CCR2^+^ cells were primarily F4/80^+^Ly6G^+^ macrophages (Figure S3c, Supporting Information). FAPs sorted from normal and injured muscles were co‐cultured with bone marrow‐derived macrophages and neutrophils in the Transwell system (Figure 2h). FAPs isolated from injured muscles showed enhanced recruitment of macrophages and neutrophils compared to FAPs from normal muscles. This recruitment effect was blocked by pre‐treatment of macrophages with the CCR2/5 antagonist (Cenicriviroc Mesylate) and neutrophils with the CXCR2 antagonist (SB225002) (Figure 2i,j). These findings indicate that aFAPs secrete chemokines to recruit inflammatory cells from the circulation.

### 2.3 IL‐33/IL1RL1 Pathway Promotes the Induction of aFAPs

**Figure 3:**
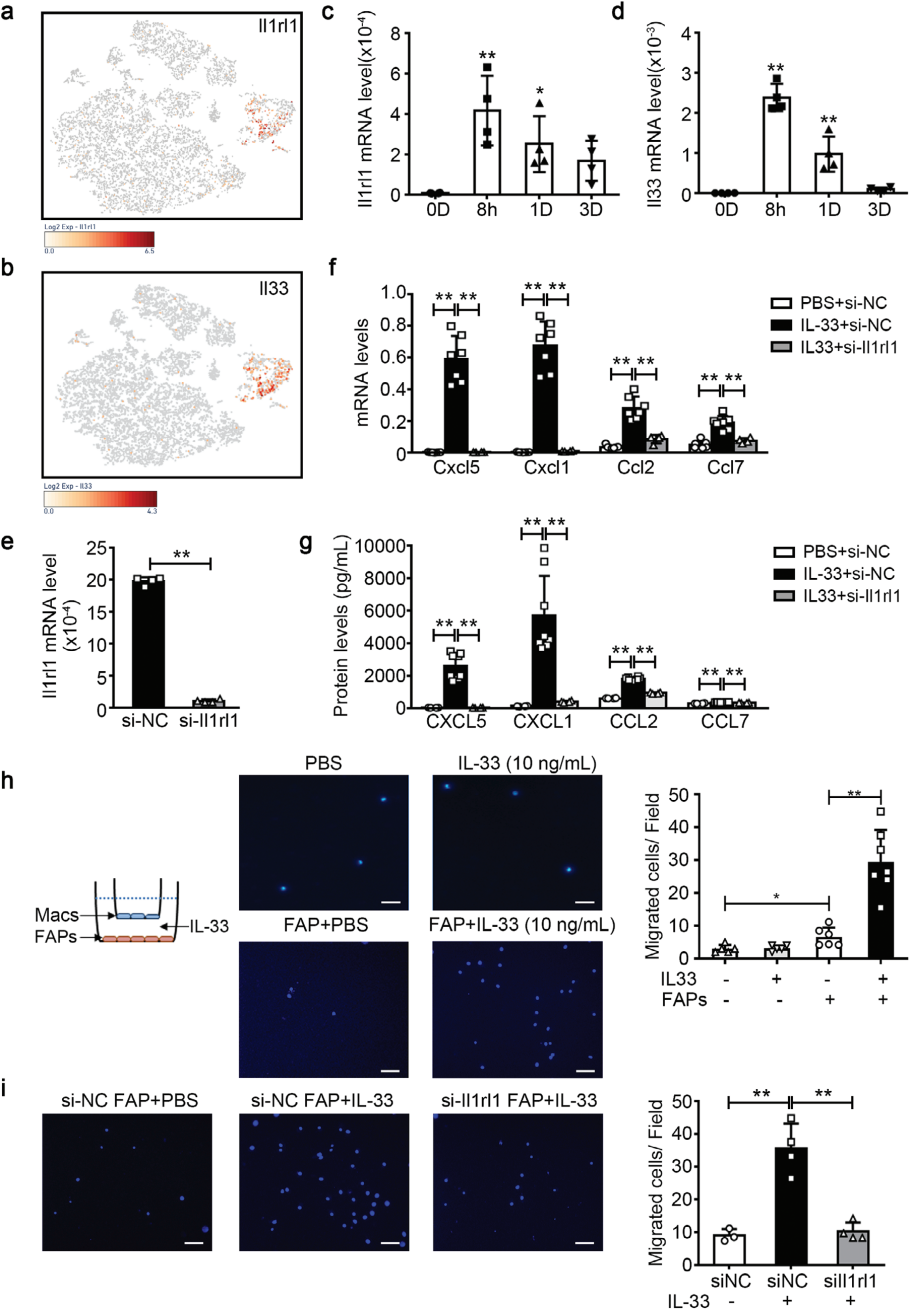
IL‐33‐IL1RL1 pathway promotes the induction of the activated FAP phenotype. a,b) The t‐SNE visualization of the cell cluster overlaid with the average expression of *Il1rl1* (a) and *Il33* (b) on day 1 after injury. c,d) The mRNA levels of *Il1rl1* (c) and *Il33* (d) in muscles on day 0 (0D), 8 hours (8h), day 1 (1D), day 3 (3D) after CTX injury were determined by qRT‐PCR (n = 4 per time‐point). e) Primary FAPs were transfected with control siRNA (si‐NC) or *Il1rl1* siRNA (si‐Il1rl1), the mRNA levels of *Il1rl1* in FAPs were determined using qRT‐PCR (n = 4 for each group). f) The mRNA levels of CXCL5, CXCL1, CCL2, and CCL7 in FAPs were analyzed using qRT‐PCR (n = 4−8 per group). g) The protein levels of CXCL5, CXCL1, CCL2, and CCL7 in the supernatant of FAPs were measured using cytokines multiplex assay (n = 4−8 per group). h) PBS, IL‐33, or PBS‐ or IL‐33‐stimulated FAPs were co‐cultured with macrophages (Macs) using the Transwell system, as shown in the illustration. The migrated macrophages per field were quantified using DAPI staining. Scale bar, 100 µm. (n = 6−7 samples per group, 10 images per sample). i) When FAPs were transfected with control siRNA or *Il1rl1* siRNA, the migrated macrophages per field were quantified using DAPI staining. Scale bar, 100 µm. (n = 3−4 samples per group, ten images per sample). Data are represented as mean ± s.e.m. For c, d, g, h, i, *p < 0.05, **p < 0.01 by one‐way ANOVA Bonferroni post‐test. For e, **p < 0.01 by by unpaired Student's *t*‐test.

Among the signature genes of the aFAP cluster, we identified *Il1rl1* as the most upregulated membrane protein‐encoding gene (Figure 2d; Table S2, Supporting Information), as confirmed by the t‐SNE map of *Il1rl1* (**Figure** **3**a). *Il1rl1* encodes the transmembrane protein, IL1RL1, which functions as an IL‐33 receptor. The t‐SNE map of *Il33* revealed its specific expression in the FAP cluster, distinct from any other cell clusters on day 1 post‐injury (Figure 3b). IL‐33 has been reported to enhance Treg cell amplification, promoting the regeneration of aging muscles.^[^ ^21^ ^]^ However, the function of the IL‐33/IL1RL1 pathway in FAPs remains unclear. qRT‐PCR data showed significantly upregulated *Il1rl1* and *Il33* mRNA levels in injured muscle as early as 8 h after injury, which decreased gradually (Figure 3c,d), preceding the upregulation of *Cxcl5*, *Cxcl1*, *Ccl2*, *Mif*, and *Ccl7* mRNA in the injured muscles (Figure S2b–f, Supporting Information). On day 1 post‐injury, the number of IL1RL1^+^ cells increased in the injured muscle, primarily composed of CD45^+^CD11b^+^ macrophages and CD45^−^CD31^−^α7‐integrin^−^Sca‐1^+^ FAPs (Figure S4a, Supporting Information).

In vitro, when FAPs were stimulated with adenosine triphosphate (ATP), a common damage‐associated molecular pattern (DAMP), the *Il33* and *Il1rl1* mRNA levels in FAPs gradually increased (Figure S4b, Supporting Information). In addition, the IL‐33 protein levels in the culture medium of FAPs also increased (Figure S4c, Supporting Information). Subsequently, the IL‐33/IL1RL1 pathway's function in FAPs was evaluated by stimulating primary FAPs with recombinant mouse IL‐33 (rIL‐33). The mRNA levels of *Cxcl5*, *Cxcl1*, *Ccl2*, and *Ccl7* in FAPs, and their corresponding protein levels in FAP supernatants increased significantly (Figure S4d,e, Supporting Information). *Il1rl1* expression significantly decreased following *Il1rl1* siRNA transfection (Figure 3e). Blocking the IL‐33/IL1RL1 pathway using *Il1rl1* siRNA decreased the IL‐33‐mediated upregulation of CXCL5, CXCL1, CCL2, and CCL7 in FAPs (Figure 3f,g). Moreover, the co‐culture of FAPs and macrophages in the Transwell system demonstrated that IL‐33‐stimulated FAPs recruited more macrophages than PBS‐stimulated FAPs, while IL‐33 alone could not recruit macrophages (Figure 3h). Additionally, *Il1rl1* siRNA blocking resulted in decreased recruitment of macrophages by IL‐33‐stimulated FAPs (Figure 3i).

Overall, these results suggest that the IL‐33/IL1RL1 pathway promotes the induction of the aFAP phenotype.

### 2.4 MAPKp38/NF‐κB Pathway Mediates the Activation of FAPs by IL‐33

**Figure 4:**
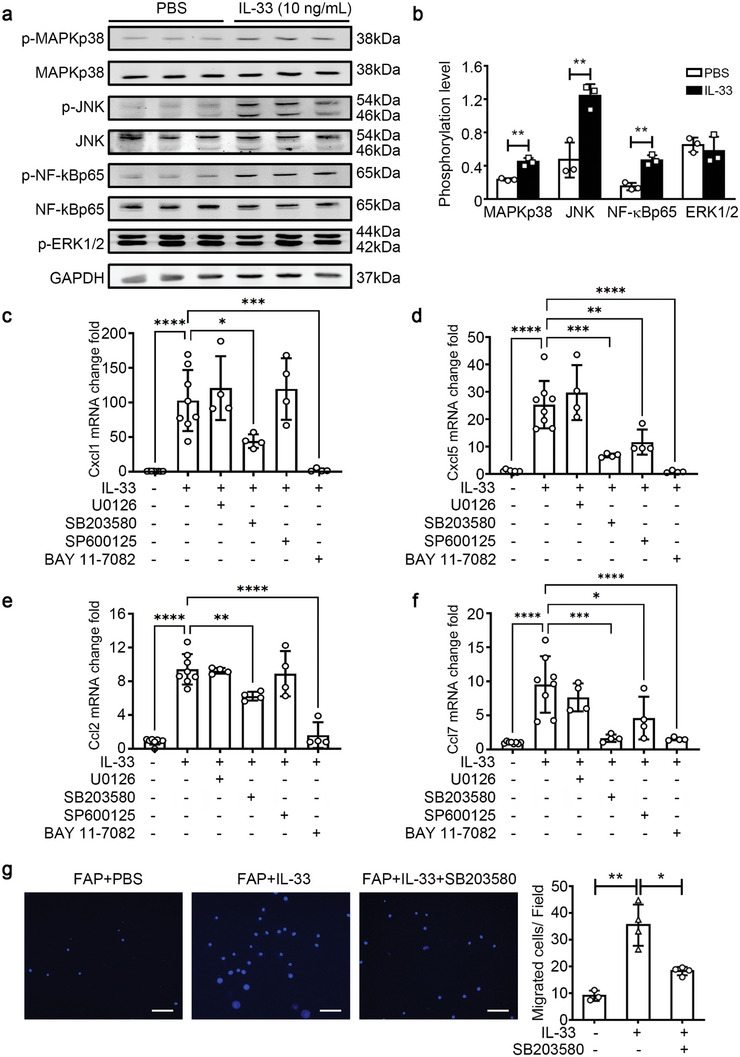
MAPKp38/NF‐κB signaling pathway mediates the IL‐33‐induced FAPs activation. a) The primary FAPs were treated with either PBS or IL‐33 for 30 min. Subsequently, protein levels of phospho‐MAPKp38 (p‐MAPKp38), total MAPKp38, phospho‐JNK (p‐JNK), total JNK, phospho‐NF‐κBp65 (p‐NF‐kBp65), total NF‐κBp65, and phospho‐ERK1/2 (p‐ERK1/2) were quantified using western blotting and normalized to GAPDH levels. b) The histogram shows the ratio of phosphorylated protein to total protein (n = 3 per group). c–f) The corresponding inhibitors of ERK1/2 (U0126), MAPKp38 (SB203580), JNK (SP600125), and NF‐κB (BAY 11–7082) were added to the culture medium of FAPs at 30 min before IL‐33 stimulation, and then the mRNA levels of *Cxcl1* (c), *Cxcl5* (d), *Ccl2* (e), *Ccl7* (f) in FAPs were determined using qRT‐PCR (n = 4 per group). g) When FAPs were pre‐treated with the MAPKp38 inhibitor SB203580 at 30 min before IL‐33 stimulation using the Transwell system, the migrated macrophages per field were quantified using DAPI staining. Scale bar, 100 µm. (n = 3−4 samples per group, ten images per sample). Data are represented as mean ± s.e.m. For b, **p < 0.01 by unpaired Student's *t*‐test. For (c–g), *p < 0.05, **p < 0.01 by one‐way ANOVA Bonferroni post‐test.

To understand the intracellular signaling pathway of IL‐33/IL1RL1 during FAP activation, we examined the phosphorylation levels of MAPKp38, JNK, NF‐κB, and ERK1/2, implicated in immune regulation,^[^ ^24^ ^]^ using western blot. We observed that rIL‐33 increased the phosphorylation levels of MAPKp38, JNK, and NF‐κB in FAPs, while ERK1/2 phosphorylation level remained unaffected (**Figure** **4**a,b). Additionally, the corresponding inhibitors of MAPKp38 (SB203580), JNK (SP600125), NF‐κB (BAY 11–7082), and ERK1/2 (U0126) were used to block the IL‐33/IL1RL1 intracellular signaling pathway. In IL‐33‐stimulated FAPs, MAPKp38 and NF‐κB inhibitors blocked the upregulation of CXCL5, CXCL1, CCL2, and CCL7 by IL‐33, while the JNK inhibitors affected the expression of these chemokines slightly and ERK1/2 inhibitors failed to affect the expression of these chemokines (Figure 4c–f; Figure S5a‐d, Supporting Information). Subsequent FAP‐mediated recruitment of macrophages was also significantly reduced by the MAPKp38 inhibitor (Figure 4g). These results showed that the activation of the MAPKp38/NF‐κB pathway contributed to the IL‐33‐induced activation of pro‐inflammatory FAPs.

### 2.5 IL1RL1 is Essential for Leukocyte Infiltration In Vivo after Injury

**Figure 5:**
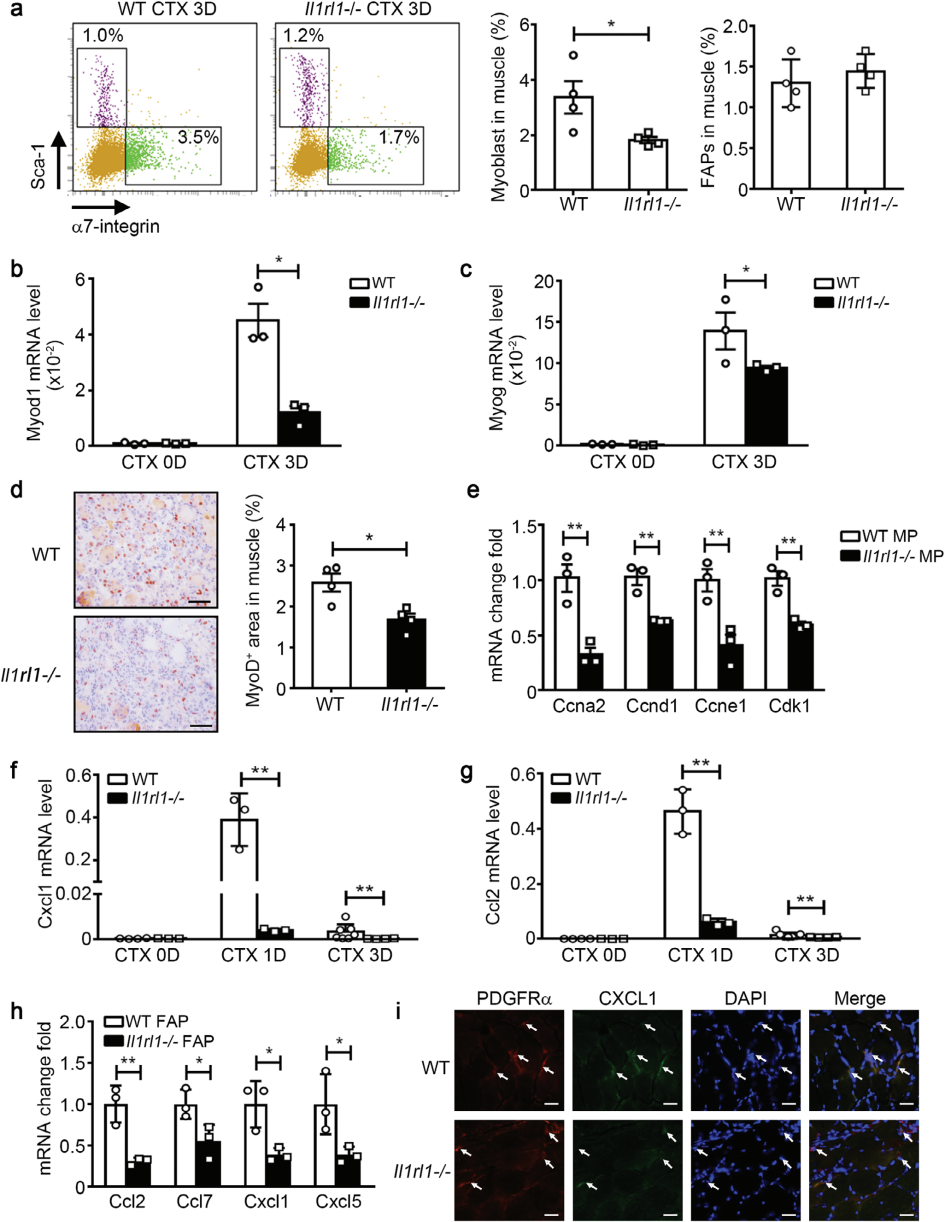
IL1RL1 is essential for FAP activation after injury in vivo. a) The ratio of CD45^−^CD31^−^α7‐integrin^+^Sca‐1^−^ MuSCs and CD45^−^CD31^−^α7‐integrin^−^Sca‐1^+^ FAPs in WT and *Il1rl1*‐/‐ muscles on day 3 post‐injury were accessed using flow cytometry (n = 4 mice per group). b,c) The mRNA levels of *Myod1* (b) and *Myog* (c) in WT and *Il1rl1*‐/‐ muscles on days 0 and 3 post‐injury were determined using qRT‐PCR (n = 3−4 mice per group). d) IHC staining and positive area quantitation of the MuSC marker MyoD in WT and *Il1rl1*‐/‐ muscles at 3 days post‐injury. Scale bars, 50 µm (n = 4 mice per group). e) CD45^−^CD31^−^α7‐integrin^+^Sca‐1^−^ MuSCs were sorted from WT and *Il1rl1*‐/‐ muscles on day 3 post‐injury, and then the mRNA levels of *Ccna2*, *Ccnd1*, *Ccne1*, and *Cdk1* in MuSCs were accessed using qRT‐PCR (n = 3 samples per group). f,g) The mRNA levels of *Cxcl1* (f) and *Ccl2* (g) in WT and *Il1rl1*‐/‐ muscles on days 0, 1, and 3 post‐injury were determined using qRT‐PCR (n = 3−4 mice per group). h) CD45^−^CD31^−^α7‐integrin^−^Sca‐1^+^ FAPs were sorted from WT and *Il1rl1*‐/‐ muscles on day 1 post‐injury. Subsequently, the mRNA levels of Ccl2, *Ccl7*, *Cxcl1*, and *Cxcl5* in FAPs were accessed using qRT‐PCR (n = 3 samples per group). i) Immunofluorescence co‐staining of PDGFRα (red) and CXCL1 (green) in WT and *Il1rl1*‐/‐ muscles on day 1 post‐injury. Scale bar, 25 µm. Data are represented as mean ± s.e.m. *p < 0.05, **p < 0.01 by unpaired Student's *t*‐test.

Next, we examined the in vivo function of the IL‐33/IL1RL1 pathway using *Il1rl1*‐/‐ mice. Using flow cytometry analysis, we observed a significantly lower number of CD45^−^CD31^−^α7‐integrin^+^ MuSCs in *Il1rl1*‐/‐ mouse muscles than in the wildtype (WT) muscles on day 3 post‐injury, whereas the ratio of CD45^−^CD31^−^Sca‐1^+^ FAPs showed no difference (**Figure** **5**a). The mRNA levels of *Myod1* and *Myog*, genes associated with MuSC proliferation and differentiation, were lower in *Il1rl1*‐/‐ mice than in WT mice (Figure 5b,c). Additionally, we examined the number of MuSCs in injured muscles using MyoD IHC staining, and the results showed a consistent trend (Figure 5d). Thereafter, MuSCs from the WT and *Il1rl1*‐/‐ muscles on day 3 after injury were sorted and examined for the expression level of proliferation‐associated genes. We discovered significantly lower mRNA levels of *Ccna2*, *Ccnd1*, *Ccne1*, and *Cdk1* in *Il1rl1*‐/‐ MuSCs than in WT MuSCs (Figure 5e), indicating the impaired proliferative ability of *Il1rl1*‐/‐ MuSCs. Moreover, the mRNA levels of *Cxcl1* and *Ccl2* were also lower in *Il1rl1*‐/‐ mice than in WT mice on days 1 and 3 post‐injury (Figure 5f,g). Then, we sorted FAPs from WT and *Il1rl1*‐/‐ muscle on day 1 post‐injury, and observed that the mRNA levels of *Ccl2*, *Ccl7*, *Cxcl1*, and *Cxcl5* in *Il1rl1‐/‐* FAPs were significantly downregulated (Figure 5h). Simultaneously, the immunofluorescence staining results also showed that the fluorescence signal of CXCL1 in PDGFRα^+^ FAPs of *Il1rl1‐/‐* skeletal muscles was relatively weak on day 1 post‐injury (Figure 5i).

**Figure 6:**
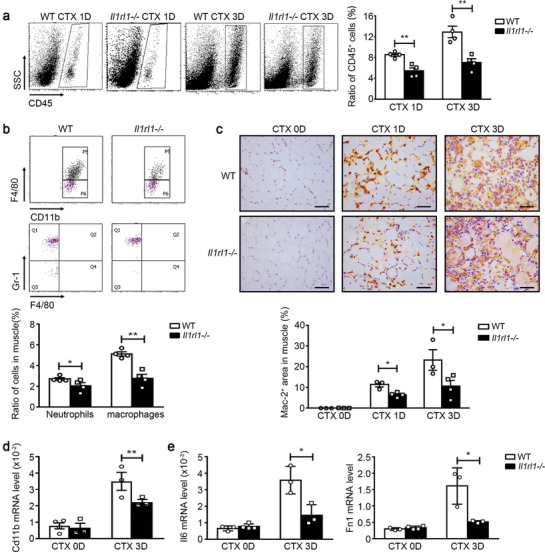
IL1RL1 is essential for leukocyte infiltration after injury in vivo. a) The ratios of CD45^+^ leukocytes in WT and *Il1rl1*‐/‐ muscles at days 1 and 3 after CTX injury were accessed using flow cytometry (n = 4 mice per group). b) The ratios of CD45^+^CD11b^+^F4/80^+^ macrophages and CD45^+^CD11b^+^F4/80^−^Gr‐1^+^ neutrophils in WT and *Il1rl1*‐/‐ muscles on day 1 after CTX injury were accessed using flow cytometry (n = 4 mice per group). c) IHC staining and positive area quantitation of the macrophage marker Mac‐2 in WT and *Il1rl1*‐/‐ muscles at days 0, 1, and 3 after CTX injury. Scale bar, 50 µm. (n = 3−4 mice per group). d,e) The mRNA levels of *Cd11b* (d), *Il6*, and *Fn1* (e) in WT and *Il1rl1*‐/‐ muscles at days 0 and 3 after CTX injury were accessed using qRT‐PCR (n = 3−4 mice per group). Data are represented as mean ± s.e.m. *p < 0.05, **p < 0.01 by unpaired Student's *t*‐test.

Furthermore, significantly fewer infiltrating CD45^+^ leukocytes in *Il1rl1*‐/‐ mouse muscles were observed than that in WT muscles on days 1 and 3 post‐injury (**Figure** **6**a). The number of CD45^+^CD11b^+^F4/80^+^ macrophages and CD45^+^CD11b^+^F4/80^−^Gr‐1^+^neutrophils decreased in the muscles of *Il1rl1*‐/‐ mice on day 1 post‐injury (Figure 6b). We also examined the infiltration of macrophages into injured muscles using immunohistochemistry (IHC) staining for galectin‐3 (also known as Mac‐2), and the results showed a consistent trend (Figure 6c). The mRNA expression levels of *CD11b* and inflammatory cell‐derived pro‐regeneration cytokines (IL‐6 and fibronectin) were also reduced in *Il1rl1*‐/‐ muscles on day 3 post‐injury (Figure 6d,e). These results indicate that the IL‐33/IL1RL1 pathway is essential for leukocyte infiltration in vivo after injury.

### 2.6 Loss of IL1RL1 Impairs Muscle Regeneration and Increases Muscle Matrix Deposition

**Figure 7:**
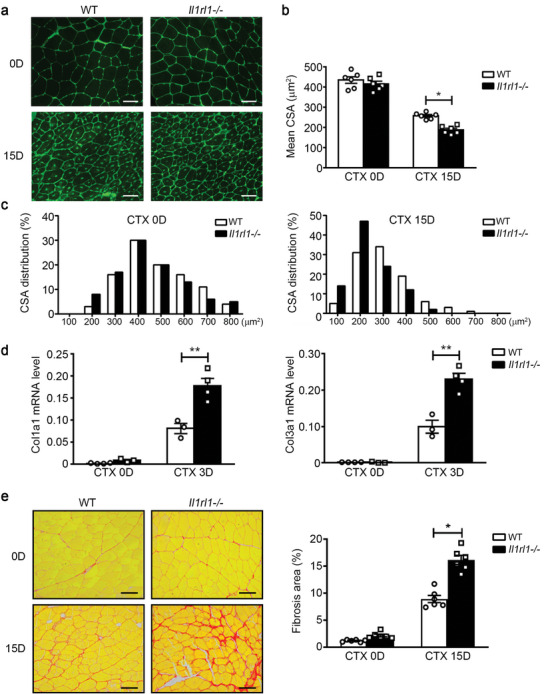
Loss of IL1RL1 impairs muscle regeneration and increases matrix deposition after injury. a) On days 0 and 15 after injury, muscles from WT and *Il1rl1*‐/‐ mice were immune‐stained with WGA (green). Scale bar, 50 µm. b) The cross‐section area (CSA) of 200−300 myofibers per muscle was examined, and the mean CSA of myofibers in WT and *Il1rl1*‐/‐ muscles was calculated (n = 6 mice per group). c) The distribution of myofiber CSA of WT and *Il1rl1*‐/‐ mice at days 0 and 15 after injury. d) The mRNA levels of Col1a1 and Col3a1 in WT and *Il1rl1*‐/‐ muscles at 0 and 3 days after CTX injury were accessed using qRT‐PCR (n = 4 mice per group). e) Picrosirius red staining was used to detect the fibrosis formation in WT and *Il1rl1*‐/‐ muscles at 0 and 15 days after CTX injury. Scale bars, 50 µm. (n = 6 mice per group). Data are represented as mean ± s.e.m. *p < 0.05, **p < 0.01 by unpaired Student's *t*‐test.

Subsequently, newly formed myofibers during late‐stage muscle regeneration were examined using wheat‐germ agglutinin (WGA) staining. There was no difference in the myofiber cross‐section area (CSA) between the WT and *Il1rl1*‐/‐ muscles before injury. However, the CSA of the newly formed myofibers in *Il1rl1*‐/‐ muscle was significantly smaller than that in WT mice on day 15 after CTX injury (**Figure** **7**a,b). Additionally, the CSA distribution of *Il1rl1*‐/‐ myofibers was considerably lower than that of the WT myofibers (Figure 7c). Additionally, Picrosirius red staining revealed increased interstitial matrix deposition in injured *Il1rl1*‐/‐ muscles (Figure 7d). Moreover, we demonstrated an association between the mRNA gene expression levels of *Col1a1* and *Col3a1* with the ECM, which were significantly higher in *Il1rl1*‐/‐ mice than in WT mice (Figure 7e). Altogether, these results suggest that the IL‐33‐IL1RL1 pathway promotes muscle regeneration.

## 3 Discussion

MuSCs and niche cells regulate skeletal muscle regeneration. However, the interactions between MuSCs and niche cells remain unclear. Two novel observations are presented in this study: 1) An activated phenotype of FAPs has been identified that chemoattracts leukocytes to promote the establishment of a regenerative niche following injury, and 2) a novel mechanism of the IL‐33/IL1RL1 signaling pathway has been identified that is involved in regulating skeletal muscle regeneration by promoting the induction of the aFAP phenotype.

These findings have important implications for understanding muscle FAP biology. FAPs are involved in the progression of muscle regeneration following injury and can regulate MuSC proliferation through the secretion of pro‐myogenic cytokines, such as IL‐6,^[^ ^16^ ^]^ follistatin,^[^ ^18^ ^]^ and WISP1,^[^ ^17^ ^]^ and contribute to the clearance of necrotic myofibers through phagocytosis.^[^ ^25^ ^]^ However, this altered environment may induce ectopic fat and fibrosis.^[^ ^19^ ^]^ In this study, we describe a novel FAP phenotype that secretes a large number of chemokines (CCL2, CCL7, CXCL1, MIF, and CXCL5) during the early stages after injury. A common receptor for CCL2 and CCL7 chemokines is CCR2, while for CXCL1 and CXCL5, it is CXCR2.^[^ ^23^ ^]^ These two receptors are primarily expressed on the surfaces of macrophages and neutrophils. Subsequent functional tests revealed that, compared to FAPs in uninjured muscles, FAPs sorted from injured muscles on day 1 exhibited an increased ability to recruit macrophages and neutrophils. Macrophages and neutrophils are important pro‐regenerative cells.^[^ ^26^ ^]^ CCR2 knockout leads to significantly reduced infiltration of macrophages into injured skeletal muscles and impaired skeletal muscle regeneration,^[^ ^27^ ^]^ suggesting a critical role of CCR2 ligands in macrophage recruitment and subsequent skeletal muscle regeneration. Bone marrow transplantation data have shown that both bone marrow cell‐derived CCL2 and muscle resident cell‐derived CCL2 are essential for the recruitment of circulating macrophages into injured muscles.^[^ ^6^ ^]^ However, the specific cellular localization of muscle‐derived CCL2 remains unclear. Our study identified that the primary cellular source of CCL2 in muscle cells is the aFAP cluster. CXCL1 and CXCL5 are critical chemokines involved in neutrophil recruitment.^[^ ^28^ ^]^ Low doses of estradiol can increase the expression of CXCL1 and CXCL5 in muscles to recruit more neutrophils to injured muscles and accelerate the recovery of muscle strength following a freeze injury.^[^ ^29^ ^]^ A recent report also showed that the chemokine CXCL7 deficiency in platelets blunts the recruitment of neutrophils to injured muscles, leading to unresolved tissue damage and impaired muscle regeneration.^[^ ^30^ ^]^ Therefore, in addition to the known functions of FAPs in muscle regeneration, our study highlights the pivotal role of FAPs in the establishment of a pro‐regenerative niche. A recently published research also reported an “early‐responder” subtype of muscle stromal cells (MmSCs) that spiked on day 1 and expressed a notable array of transcripts encoding immunomodulators, including IL‐33 and other chemokines. The inflammatory MmSC subtype orchestrates immunocyte recruitment to injured tissue.^[^ ^31^ ^]^ These findings are consistent with our conclusion in this study.

The expression levels of chemokines were undetectable in the muscles and serum of uninjured mice. However, their expression peaked within 24 h after the injury and then began to decline. Macrophages amplified the immunomodulatory program of activated MmSCs but were not strictly required for the induction of MmSC activation,^[^ ^31^ ^]^ and the underlying mechanism of this induction is incompletely understood. In our study, after comparing specific gene expression in the FAP clusters, we identified *Il1rl1* as the most upregulated membrane receptor molecule within the aFAP cluster. *Il1rl1* encodes the protein IL1RL1, also known as ST2, which is a receptor for the cytokine IL‐33. IL‐33, as a member of the IL‐1 family of cytokines, was first discovered to drive the production of Th2‐associated cytokines (IL‐4, IL‐5, and IL‐13) in polarized Th2 cells by activating the NF‐κB and MAPK pathways.^[^ ^32^ ^]^ Subsequent research has demonstrated that IL‐33 has immunoregulatory abilities, such as mediating mast cell activation to mobilize macrophages and promote gastric cancer ^[^ ^33^ ^]^ or activating M2‐like macrophages to promote tissue repair.^[^ ^34^ ^]^ A previous study on skeletal muscle regeneration showed that FAPs secrete a large amount of IL‐33 at a very early‐stage (6–12 h) after injury, while IL‐33 expression in FAPs in older mice was decreased.^[^ ^21^ ^]^ Administration of recombinant IL‐33 to aged mice enhanced the proliferative ability of infiltrated Treg cells in muscles and promoted MuSC proliferation and muscle regeneration.^[^ ^21^ ^]^ In this study, we observed a significant upregulation of IL‐33 and IL1RL1 mRNA levels in FAPs during the initial stage (8 h) after injury. Given that Treg cell proliferation occurs primarily 2−3 days after injury,^[^ ^35^ ^]^ this suggests that IL‐33 may serve other functions during the early stages following muscle injury.

In this study, we discovered that IL‐33, acting as an alarmin, rapidly activated the resident FAPs to secrete chemokines, recruiting macrophages and neutrophils by activating its own IL1RL1 receptor and downstream MAPKp38 signaling pathway. FAPs derived from *Il1rl1*‐/‐ muscles expressed lower chemokine levels, impairing macrophage recruitment. Additionally, we observed reduced macrophage and neutrophil infiltration in *Il1rl1*‐/‐ muscles post‐injury. A recent study explored changes in FAP function in older mice via transcriptome sequencing. Among the DEGs, the expression levels of chemokines−CCL2, CCL7, and CXCL2−in FAPs of older mice were reduced significantly,^[^ ^17^ ^]^ possibly associated with decreased IL‐33 levels in older mice. In the *Il1rl1‐/‐* mouse, we found a decrease in the infiltration of macrophages and neutrophils, but it was not completely blocked. This is because the source of chemokines responsible for recruiting inflammatory cells is not only limited to FAPs but extends beyond them. Bone marrow transplantation experiments showed that the main macrophage chemotactic factor CCL2 partially originates from bone marrow‐derived cells and partially from muscle resident cells.^[^ ^6^ ^]^ However, its origin in resident cells remains unclear. This study demonstrates that FAPs within muscles can also secrete CCL2 to recruit macrophages to the injury site. Additionally, platelet‐derived CXCL7 has been shown to promote neutrophil recruitment. In *Cxcl7*‐/‐ mice, the number of neutrophils decreased by only half, resulting in a decrease in the ability of skeletal muscle regeneration. However, it did not affect the expression of another chemotactic factor, CXCL1, responsible for recruiting neutrophils.^[^ ^30^ ^]^ In this study, we found that FAPs activated by the IL33‐IL1RL1 axis can produce a large amount of CXCL1 and recruit neutrophils to the injury site, thereby supplementing the molecular mechanism of early inflammatory cell infiltration in skeletal muscle injury.

The mRNA expression level of IL‐33 was rapidly upregulated in muscle resident FAPs within hours after injury, which is consistent with previous results.^[^ ^21^ ^]^ However, the mechanisms underlying IL‐33 upregulation remain unclear. Stimulation with TLR3 and TLR4 activators (poly I: C, LPS, and others) and the transcription factors IRF3 and CREB was shown to enhance the mRNA transcription of IL‐33 in mouse macrophages and embryonic fibroblasts.^[^ ^36^, ^37^ ^]^ Additionally, stimulation with PMA and IL‐1β in cardiac fibroblasts could upregulate the mRNA level of IL33.^[^ ^38^ ^]^ Simultaneously, bioactive IL‐33 is stored within epithelial cells, macrophages, and other cells, and extracellular ATP can directly stimulate its release.^[^ ^39^ ^]^ Necrotic myofibers release large numbers of death‐associated molecular patterns (DAMPs), such as ATP, HSP, and HMGB1, and other non‐protein components from ECM hyaluronan fragments.^[^ ^40^ ^]^ These DAMPs can activate TLRs and RAGE on the cell surface.^[^ ^41^ ^]^ In this study, we found that after being stimulated by ATP, the mRNA levels of *Il33* and its receptor, *Il1rl1*, increased. The concentration of IL‐33 protein in the culture medium of FAPs also showed a consistent trend of change. These results demonstrated that the upregulation of IL‐33 in FAPs was stimulated by DAMPs released from the damaged myofiber. However, the fold change of IL‐33 protein in culture medium after LPS stimulation was relatively lower than that of mRNA in cells, which may be attributed to the existence of complex regulation mechanisms of IL‐33 release. Current studies show that full length IL‐33 can be released from the nucleus of different cell types upon mechanical stress or other stimuli, such as nigericin or IL‐1β, even in the absence of cellular necrosis.^[^ ^42^, ^43^, ^44^ ^]^ Upon stimulation, nuclear IL‐33 appears to translocate to the cytoplasm via the nuclear pore, where it may reside in membrane‐bound vesicles to facilitate extracellular secretion from cells. Additionally, the extracellular release of IL‐33 was also mediated by perforin‐like molecules in living cells.^[^ ^45^ ^]^ Gasdermin D has been demonstrated to mediate the release of IL‐33 from senescent hepatic stellate cells.^[^ ^46^ ^]^ However, the release regulation mechanism of IL‐33 from FAPs is still unclear and requires further experimentation for confirmation.

In conclusion, impaired muscle regeneration results in muscle atrophy and decline in exercise capacity, this study underscored the critical role of muscle‐resident cells in inflammation and muscle regeneration, and found that the IL‐33/IL1RL1 axis plays a crucial role in activating FAPs to recruit pro‐regenerative inflammatory cells, which will provide a new therapeutic target for skeletal muscle atrophy caused by aging or other diseases.

## 4 Experimental Section

### Mice

All experimental procedures were performed following the institutional guidelines for animal studies and approved by the ethics committee of Capital Medical University (the approval number: 2023047X). *Il1rl1* deficient (*Il1rl1*‐/‐) mice and littermate controls (WT) in BALB/c background were obtained from Professor Andrew McKenzie ^[^ ^47^ ^]^ and bred in the animal facility of Beijing Anzhen Hospital affiliated with the Capital Medical University. Mice were housed in a specific pathogen‐free environment under constant temperature and humidity, and a 12/12 h light‐dark cycle with free access to food and water. Male *Il1rl1*‐/‐ and WT mice (10∼12 weeks) were used for in vivo experiments.

### Muscle Regeneration Model

CTX injury‐induced muscle regeneration model was established as described earlier.^[^ ^12^ ^]^ Briefly, TA and gastrocnemius muscles of the anesthetized mice (100 mg Kg^−1^ of 1% pentobarbital sodium, i.p.) were injected with 30 and 60 µL of 10 µm of CTX (Sigma), respectively. On day‐1, −3, and −14 after injury, mice were weighed and sacrificed by cervical dislocation under anesthesia. The TA muscles and gastrocnemius muscles were collected. The muscle samples were embedded in optimal cutting temperature compound, frozen in isopentane chilled with liquid nitrogen for histological analysis or frozen in liquid nitrogen for mRNA and protein extractions. For flow cytometry and other experiments, fresh muscles were used.

### Digestion of the Muscle

As described previously,^[^ ^48^, ^49^ ^]^ minced TA muscle was digested at 37 °C for 30 min in phosphate‐buffered saline (PBS) containing collagenase I (200 U mL^−1^) and dispase II (2.4 U mL^−1^), and dissociated further by the gentleMACS dissociator C tubes using the mouse‐muscle program (Miltenyi Biotec). The samples were filtered, centrifuged, and resuspended in PBS. The single‐cell suspension was used for scRNA‐seq or flow cytometry analysis.

### scRNA‐seq and Analysis

The harvested single cells were subjected to single‐cell library preparation by 10 × Genomics platform (BGI, Shenzhen, China), followed by paired‐end 100 sequencing using the Illumina HiSeq 4000. Cell Ranger v2.1.0 processed the raw data from 10 × Genomics scRNA‐seq. The cellranger mkfastq was used to demultiplex BCL files from HiSeq 4000 into FASTQs for analysis. Cellranger counts were used for mapping reads to the reference genome (GRCm38/mm10) based on STAR aligner v2.5.1b43 and to generate gene‐cell‐barcode matrices for each sample. The gene‐cell‐barcode matrices from samples were concatenated. For quantification of transcript abundance, the number of transcripts containing unique molecular identifiers (UMI) per cell‐specific barcode was counted for each gene. For the quality control and filtering of data, cells with mitochondrial gene content >20%, and low quality cells (nFeature_RNA < 200) and doubles (nFeature_RNA > 5000) were removed. Next, the Loupe Cell software was used to cluster cells based on κ‐means clustering, visualize cell clusters using uniform manifold approximation and projection (UMAP) or t‐distributed stochastic neighbor embedding (t‐SNE), and analyze differentially expressed genes (DEG) by computing up‐ or down‐regulated genes in all cells within the cluster compared to cells not in the cluster. Go function enrichment analysis was used to explore the functions of DEG of cell cluster. CellChat DB analysis was used to explore the cell‐cell communications.

### Flow Cytometry Analysis and Sorting

The harvested single cells were resuspended in PBS at 1 × 10^7^ cells mL^−1^. The cells were incubated with antibodies at 4 °C for 30 min in the dark, then washed and resuspended in PBS. All antibodies used in the flow cytometry experiments were listed in Table S3 (Supporting Information). The expression of cell surface molecules was analyzed by flow cytometry (BD LSRFortessa) analysis using the associated software (BD FACSDiva Software). For isolation of FAPs, CD45^−^CD31^−^a7‐integrin^−^Sca‐1^+^ cell was set as the sort gate for BD FACSAria III flow cytometry (BD). After sorting, 1 × 10^5^ cells per well were seeded in Matrigel‐coated 24‐well plate. The FAPs were cultured in DMEM high glucose medium supplemented with 10% fetal bovine serum (FBS), 1% penicillin‐streptomycin (PS), and 10 ng mL^−1^ recombinant mouse basic‐FGF protein (Perprotech).

### Isolation of Bone Marrow‐Derived Macrophages (BMDMs) and Neutrophils

Isolation of primary BMDMs and neutrophils was performed as described previously.^[^ ^50^ ^]^ Bone marrow cells from WT mice were flushed out from femurs and tibias using a 25G needle and filtered. Cells were centrifuged at 1000 rpm for 10 min, resuspended in fresh growth medium (DMEM high glucose medium supplemented with 10% FBS and 1% PS). For macrophages, cells were seeded in plates and all the non‐adherent cells were removed after 4 hours, adherent cells were cultured with fresh growth medium supplemented with 50 ng mL^−1^ m‐CSF (PeproTech) in a humidified 5% CO_2_ atmosphere at 37 °C. For neutrophils, 3 mL Histopaque1119, 3 mL Histopaque 1077 (Sigma) and 3 mL bone marrow cells suspension were added to 15 mL centrifuge tubes in sequence., and were centrifuged at 2000 rpm for 20 min at 4 °C. Collected the neutrophils at the interface of the Histopaque 1119 and Histopaque 1077 layers using a pipet. After transferred to a new 15 mL centrifuge tube, cells were diluted with PBS solution and were centrifuged at 1500 rpm for 10 min at 4 °C. After discarding the supernatant, cells were resuspended with 1640 medium with 10% FBS and 1% PS. The fresh isolated neutrophils were used for transwell migration assay.

### mRNA Extraction and Quantitative Realtime‐PCR (qRT‐PCR)

Total mRNA was extracted from muscles or cells using TRIzol reagent (Invitrogen, Carlsbad, CA) as described previously.^[^ ^49^ ^]^ The mRNA concentrations were measured using the Nanodrop ND‐2000C (Thermo Scientific). The 5 µg mRNAs of each sample were reverse transcribed using the GoScript Reverse Transcription kit (Promega, Madison, WI). qRT‐PCR was performed with a 2x SYBR master mix (Takara), using a BIO‐RAD CFX CONNECT system (Bio‐Rad). The primers for each gene are presented in Table S4 (Supporting Information). Data were presented as values calculated by 2^−DDCt^ method with β‐Actin as the housekeeping gene.

### Protein Extraction and Western Blot

Proteins from cells were extracted with protein lysis buffer (Thermo Fisher, Waltham, MA) and incubated on ice for 30 min. After centrifugation at 12,000 rpm for 15 min, the supernatants were collected. Protein concentrations were determined by the BCA protein assay kit (Thermo Fisher). Approximately 30 µg of protein sample was separated by sodium dodecyl sulfate‐polyacrylamide gel electrophoresis (SDS‐PAGE) and transferred to nitrocellulose membranes (PALL BioTrace). After blocking in 5% BSA, membranes were incubated overnight at 4 °C with the primary antibody diluted in TBST buffer. Later, the membranes were incubated for 1 h at room temperature in the dark with TBST diluted infrared Dye 800‐conjugated secondary antibodies (Li‐COR Biosciences). All antibodies and dilutions used are listed in Supplementary Table S3. Images were acquired and analyzed by the Odyssey infrared imaging system (Li‐COR Biosciences). The protein expression level was corrected using GAPDH as a control.

### Histological Analysis

Serial, transverse cryosections (7 µm thick) of frozen TA muscles from the mid‐belly region were obtained by slicing at −20 °C using a CM1950 Frigocut (Leica). All cryosections were kept at −80 °C until further studies. The cross‐sectional area (CSA) of myofibers was analyzed by immunostaining the sections with FITC‐conjugated WGA (1:200 diluted, Sigma). Picrosirius red staining was used to detect collagen deposition. The uninjured muscles (day‐0) were used as controls. For IHC staining, the sections were first rehydrated with 1x phosphate‐buffered saline buffer (PBS) and fixed with 4% paraformaldehyde for 10 min, then blocked with 10% BSA at room temperature (RT) for 30 min. The primary antibodies were diluted in 1x PBS and added for overnight incubation at 4 °C. After a washing step with PBS, the corresponding secondary antibodies were added and incubated for 30 min at RT and stained with 3, 3′‐diaminobenzidine (DAB). All antibodies used for IHC staining are listed in Table S3 (Supporting Information). Images obtained from each muscle section (ECLIPSE 90i, Nikon) were analyzed by NIS‐Elements Br 3.0 software. For analysis of the CSA of myofibers, 200–300 myofibers per sample were examined.

### Mouse 36 Cytokines Multiplex Assay

The concentrations of cytokines and chemokines in the mouse serum and cell culture medium were analyzed by the 36‐Plex Mouse ProcartaPlex Assay Kit (Thermo Fisher) following the manufacturer's instructions.

### IL33 ELISA Assay

The culture medium of FAPs was collected at 0, 4, 8, and 12 h after ATP stimulation, then the concentration of IL‐33 was analyzed by IL‐33 ELISA kit (Invitrogen, BMS6025) following the manufacturer's instructions.

### Cell Migration Analysis

Cell migration was quantitated in duplicate using the 24‐well Transwell inserts with polycarbonate membrane (8.0 µm pore size) (Corning Costar). Macrophages or neutrophils (1 × 10^4^ per well) were added to the upper chamber of the insert. For neutrophils, 5ug mL^−1^ fibronectin was used to coat upper chambers to enhance the cell adhesion to the membrane. In some conditions, macrophages were pre‐treated with CCR2/CCR5 antagonist Cenicriviroc Mesylate (1 µm), and neutrophils were pre‐treated with CXCR2 antagonist SB225002 (500 nm) for 30 min before co‐culture. The lower chambers were seeded with FAPs (1 × 10^5^ per well), which were stimulated with PBS or IL‐33 (10 ng mL^−1^, R&D Systems) and MAPKp38 inhibitors SB203580 (10 µm). After co‐cultured for 12 h at 37 °C, the macrophages or neutrophils were fixed using 4% formalin for 15 min, the inner side cells were wiped off by cotton swabs, and cells that had migrated to the outer side of upper chamber membrane were stained with DAPI. The migrated cells of each transwell membrane were photographed by microscope (Nikon) for 10‐fields and the number of migrating cells in each field were counted.

### Statistical Analysis

Unless otherwise stated, data are represented as mean ± s.e.m. Unpaired two‐tailed Student's *t*‐test was used to assess statistical differences between two groups, and statistical differences between multiple groups were evaluated by one‐way ANOVA Bonferroni post‐test. Details of the *n* number can be found in the appropriate figure legend. All data were analyzed by GraphPad Prism software (version 9.0). For all statistical tests, *p* < 0.05 is considered statistically significant. Detailed statistical value can be found in the figure legends.

## Conflict of Interest

The authors declared no conflict of interest.

## Author Contributions

C.Z., G.L., and F.Z. contributed equally to this work. C.Z. and Y.L. initiated and managed the project; C.Z., G.L., and F.Z. designed and conducted experiments and analyzed data. Y.Z. performed data analysis of scRNA‐seq. F.Z. and S.H. participated in mouse model and pathologic analysis. Y.L., S.G., and J.D. discussed the results and commented on the manuscripts. C.Z. and Y.L. wrote and edited the manuscript. All authors reviewed the manuscript.

## Supporting information

Supporting Information

## Data Availability

The data that support the findings of this study are available from the corresponding author upon reasonable request.
