# Supplementary material for: IL‐33 Facilitates Fibro‐Adipogenic Progenitors to Establish the Pro‐Regenerative Niche after Muscle Injury
Source: Adv Sci (Weinh). 2024 Jul 22;11(35):2405299. doi: 10.1002/advs.202405299 (PMC11425282; doi:10.1002/advs.202405299)
Supplement: Supplementary file 1 — Supporting Information [file ADVS-11-2405299-s001.docx]

Supplemental Information

**IL-33 Facilitates Fibro-adipogenic Progenitors to Establish the Pro-regenerative Niche After Muscle Injury**

*Congcong Zhang^#,^ *, Guoqi Li^#^, Fan Zhang^#^, Yanhong Zhang, Shiyao Hong, Shijuan Gao, Yan Liu, Jie Du, Yulin Li**

^#^ These authors contributed equally to this study.


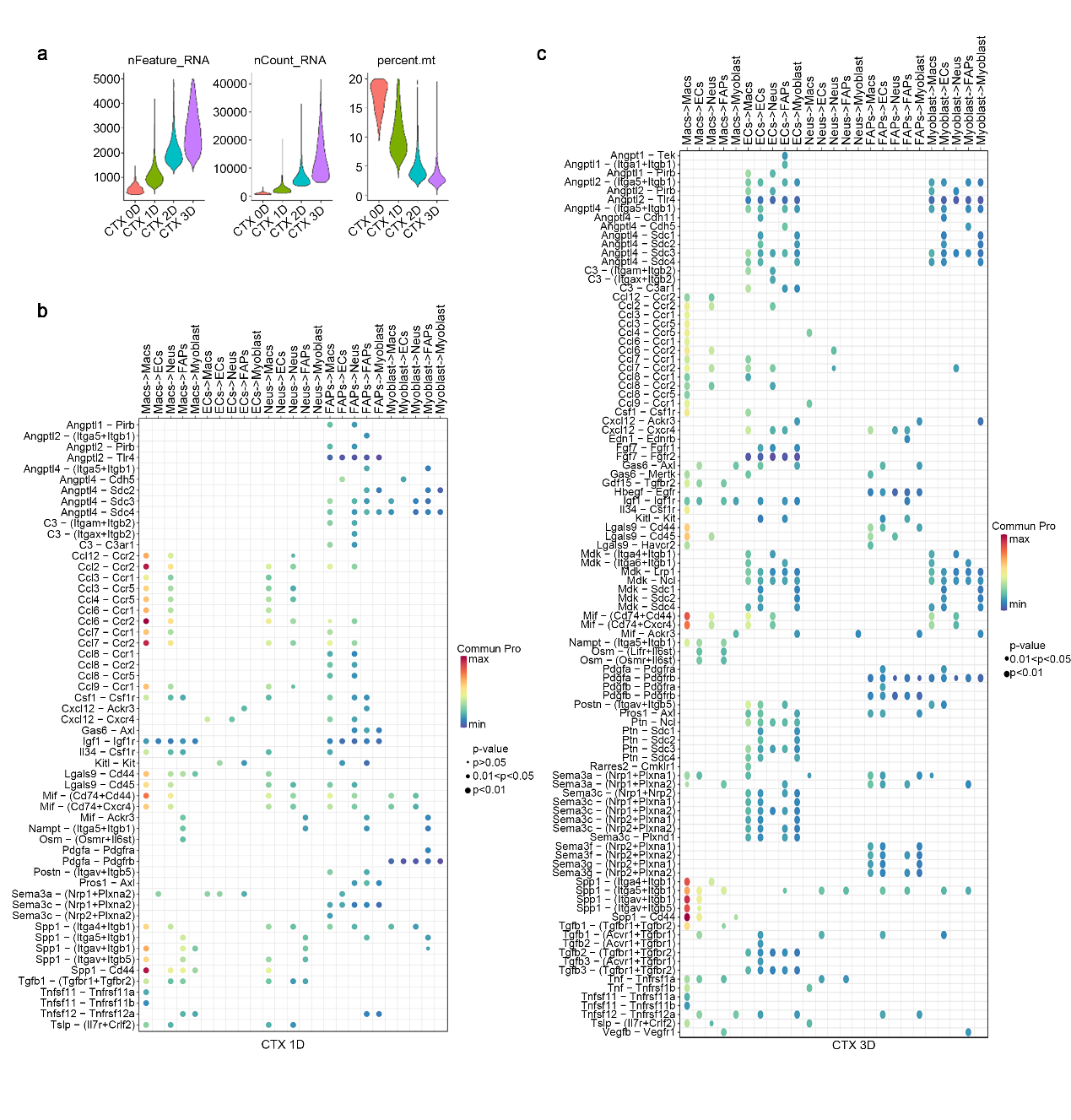


**Figure S1.** a) The samples information filtered by quality control**.** b-c) Cell-cell communication analysis by Cellchat DB. The Ligand and receptor pairs between different cell types at day-1 (b) and day-3 (c) were listed.


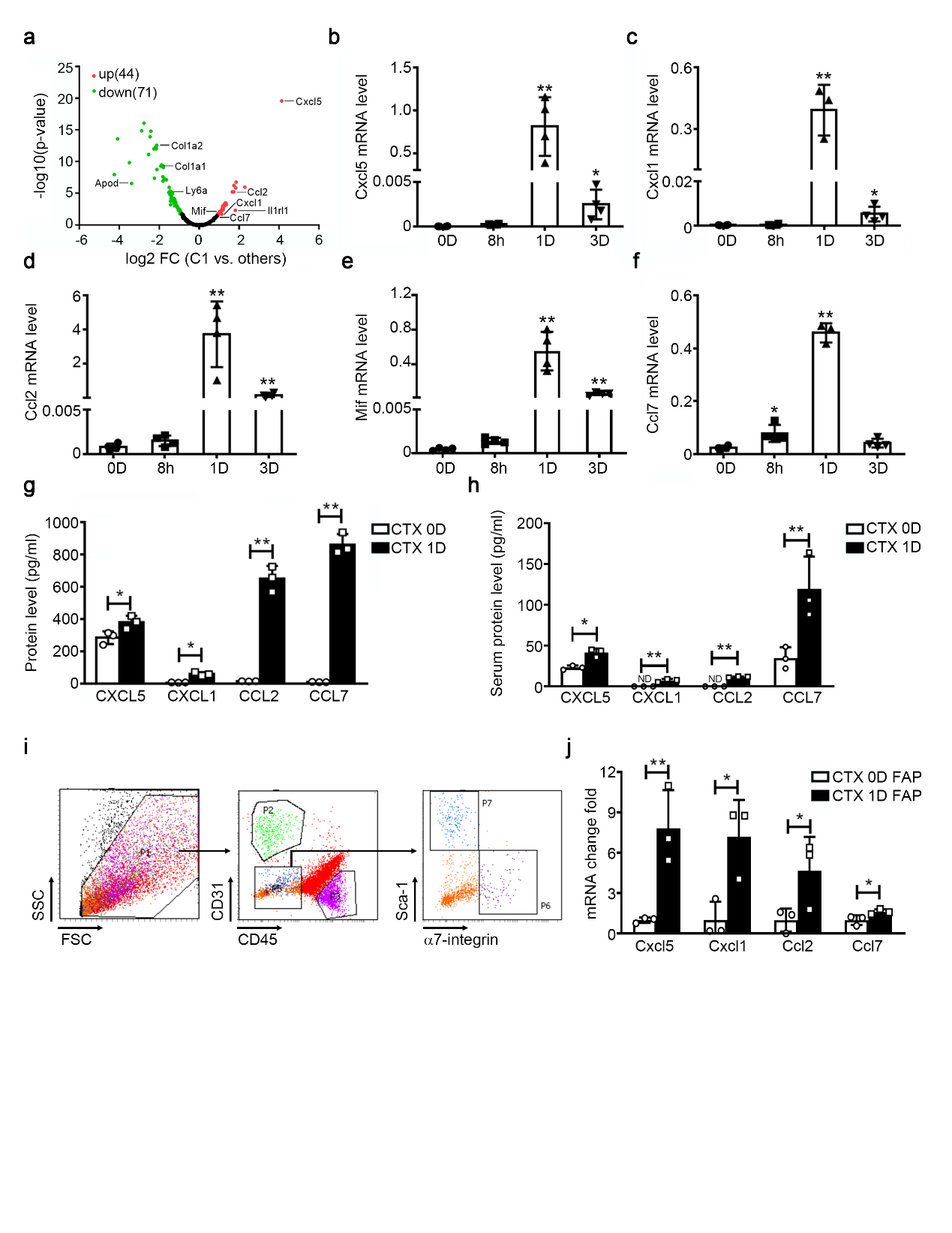


**Figure S2.** Activated FAPs secrets chemokines. a) Volcano plot of gene expression fold change between C1 FAP cluster and other two cluster (x axis, log2 fold change; y axis, p value significance). p values were determined by Mann-Whitney U test with FDR correction. b-f) The mRNA levels of *Cxcl5* (b), *Cxcl1*(c), *Ccl2* (d), *Mif* (e), *Ccl7* (f) in muscles from 0 hour (0D), 8 hours (8h), 24 hours (1D), 72 hours (3D) after CTX injury were determined by qRT-PCR (n=4 mice per time point). g) The protein levels of CXCL5, CXCL1, CCL2, CCL7 in muscles from day-0 and day-1 after CTX injury were measured by cytokines multiplex assay (n=3 mice per group). h) The protein levels of CXCL5, CXCL1, CCL2, CCL7 in muscles from day-0 and day-1 after CTX injury were measured by cytokines multiplex assay (n=3 mice per group). i) The gating strategy for FACS sorting FAPs from muscle. j) The mRNA level change fold of *Cxcl5*, *Cxcl1*, *Ccl2* and *Ccl7* in sorted FAPs was determined by qRT-PCR (n=3 mice per group). Data are represented as mean ± s.e.m. For b-f, *p<0.05, **p<0.01 relative to 0 day by One-way ANOVA Bonferroni post-test. For g, h, j, *p<0.05, **p<0.01 relative to day-0 by unpaired Student’s *t*-test.


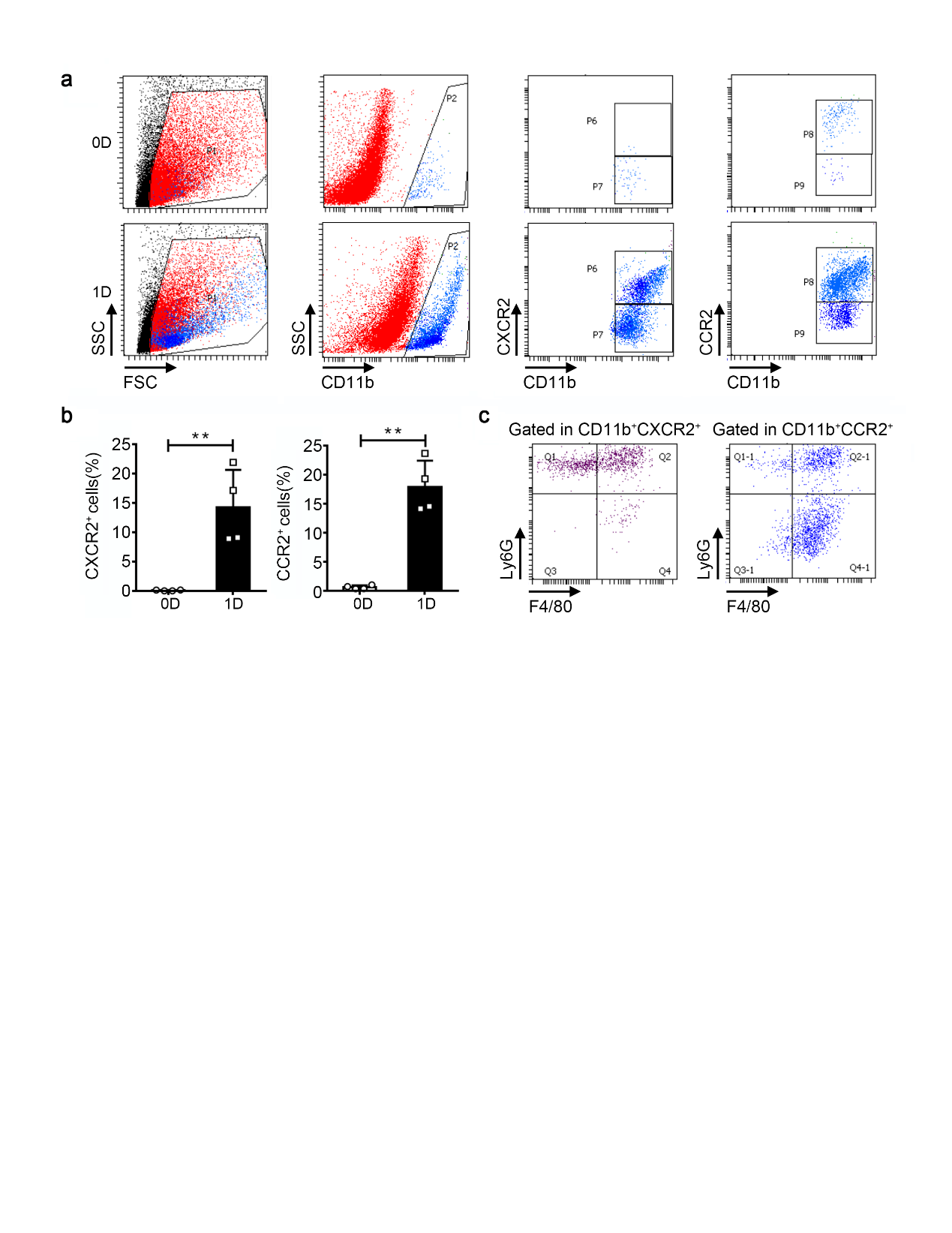


**Figure S3.** CXCR2^+^ and CCR2^+^ cells were macrophages and neutrophils. a) CD11b^+^CXCR2^+^ cells and CD11b^+^CCR2^+^ cells in muscles from day-0 and day-1 after CTX injury were examined by flow cytometry. b) The ratio of CD11b^+^CXCR2^+^ cells and CD11b^+^CCR2^+^ cells in muscle was analyzed (n=4 mice per group). c) The ratio of F4/80^+^Gr1^hi^, F4/80^+^Gr1^low^ macrophages and F4/80^-^Gr1^hi^ neutrophils in CD11b^+^ CXCR2^+^ cells and CD11b^+^CCR2^+^ cells was analyzed. Data are represented as mean ± s.e.m. For b, **p<0.01 by unpaired Student’s *t*-test.


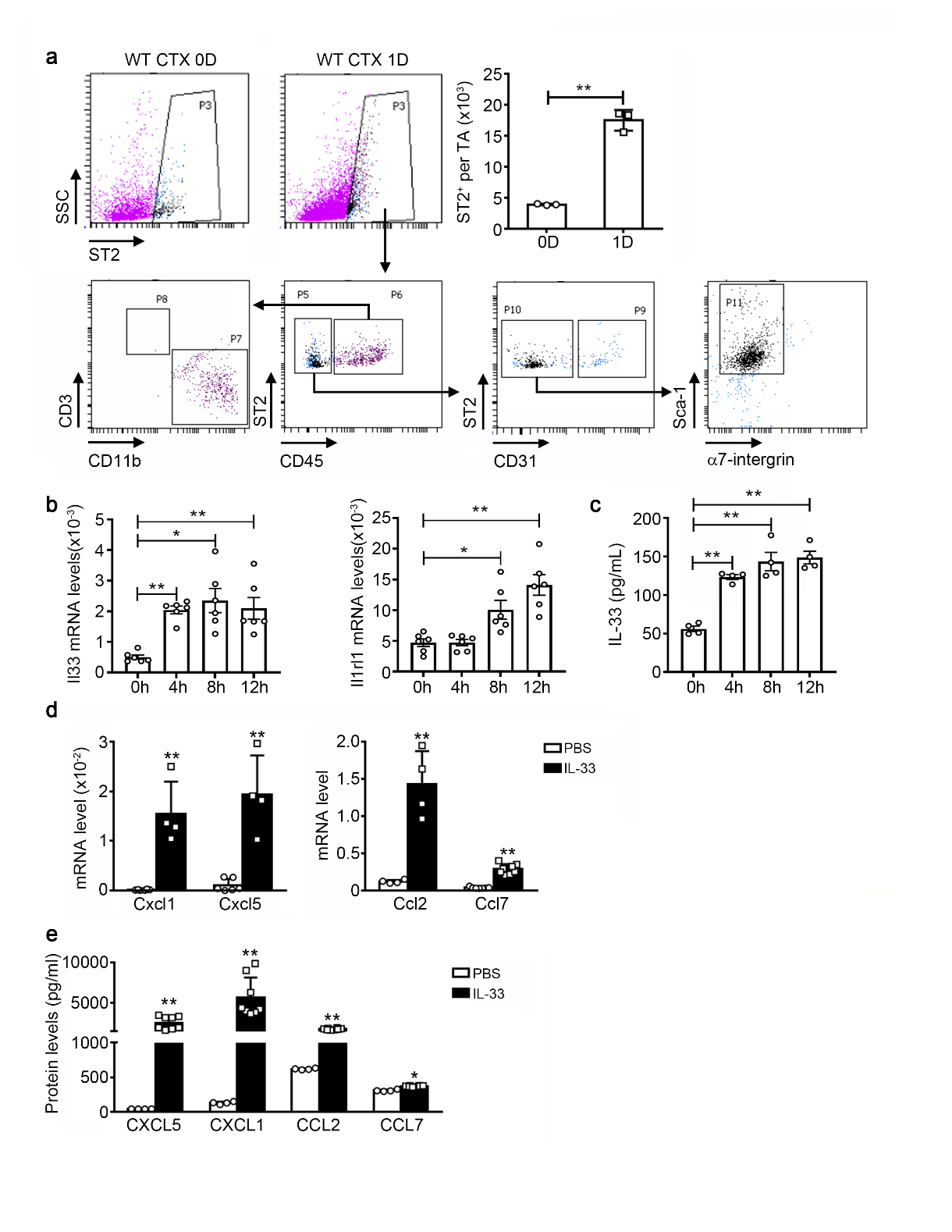


**Figure S4.** IL-33/IL1RL1 contributes to the differentiation of aFAP. a) The ratio of ST2^+^ cells in muscles at 0 day and 1 day after CTX injury were examined by FACS. Then the ratio of ST2^+^CD45^+^ and ST2^+^CD45^-^ cells in ST2^+^cells, the ratio of CD11b^+^ monocyte and CD3^+^ lymphocyte in ST2^+^CD45^+^ cells, and the ratio of CD31^+^ endothelia cells, CD31^-^a7-integrin^+^ myoblast and CD31^-^a7-integrin^-^Sca-1^+^ FAPs in ST2^+^CD45^-^cells was also analyzed by FACS. b) The mRNA levels of Il33 and Il1rl1 in FAPs with ATP stimulation for 0, 4, 8 and 12 hours were accessed by qRT-PCR. c) The concentrations of IL-33 in the FAPs culture medium were accessed by ELISA. d) The mRNA levels of *Cxcl5*, *Cxcl1*, *Ccl2*, and *Ccl7* in FAPs with PBS (n=4) or recombinant IL-33 (n=4) stimulation were accessed by qRT-PCR. e) The protein levels of CXCL5, CXCL1, CCL2, CCL7 in supernatant of FAPs with PBS (n=4) or recombinant IL-33 stimulation (n=8) were accessed by Mouse 36 cytokines multiplex kit. Data are represented as mean ± s.e.m. For b, c, *p<0.05, **p<0.01 by One-way ANOVA Bonferroni post-test. For d, e, *p<0.05, **p<0.01 by unpaired Student’s *t*-test.


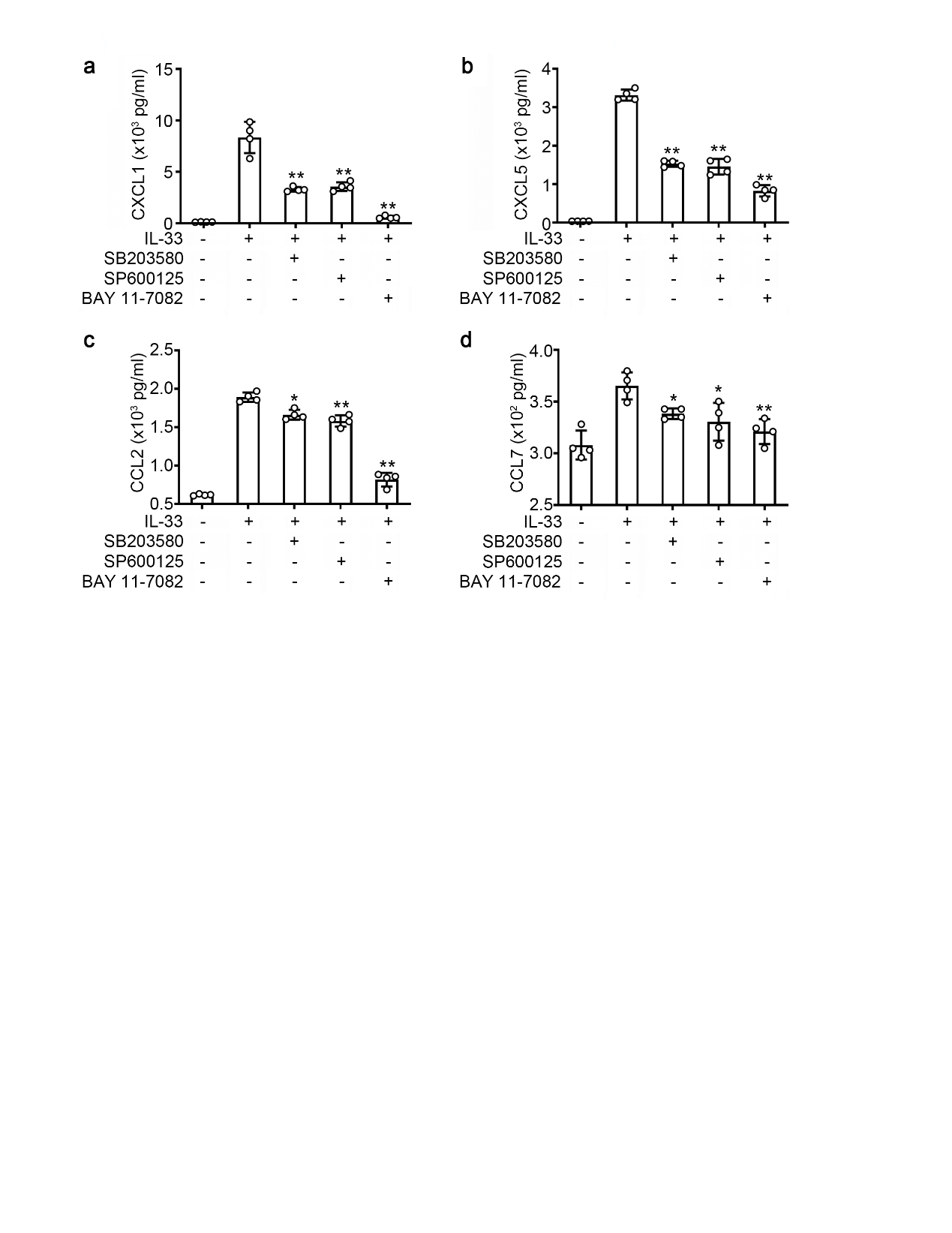


**Figure S5.** MAPKp38 pathway mediates the IL-33 induced aFAPs differentiation. a-d) The corresponding inhibitors of MAPKp38 (SB203580), JNK (SP600125), NF-κB (BAY 11-7082) were added to the culture medium of FAPs at 30 min before IL-33 stimulation, then the protein levels of CXCL1 (a), CXCL5 (b), CCL2 (c), CCL7 (d) in FAPs were accessed by cytokines multiplex assay (n=4 sample per group). *p<0.05, **p<0.01 relative to IL-33 group by one-way ANOVA Bonferroni post-test.

**Table S1. DEGs in the C1 clusters of FAPs. (****P-value<0.05)**

| **Feature Name** | **C1 Average**  **UMI** | **C2 and C3 Average**  **UMI** | **Log2 Fold Change (C1 vs C2)** | **P-Value** |
| --- | --- | --- | --- | --- |
| Cxcl5 | 10.58071415 | 0.601325 | 4.132722888 | 2.69E-20 |
| Serpine1 | 1.237834974 | 0.2513 | 2.287238279 | 1.06E-06 |
| Hspd1 | 2.046167754 | 0.565425 | 1.85132399 | 1.76E-07 |
| Nop58 | 1.124427091 | 0.314125 | 1.830221565 | 1.44E-06 |
| Il1rl1 | 1.457411938 | 0.41285 | 1.813066205 | 0.005043 |
| Ncl | 3.507199093 | 1.050075 | 1.738788684 | 5.81E-07 |
| Ccl2 | 6.558353722 | 1.977492 | 1.730316455 | 5.93E-06 |
| Nhp2 | 1.245073775 | 0.391908 | 1.660639168 | 6.17E-06 |
| Cd44 | 1.625110829 | 0.631242 | 1.361093076 | 0.000595 |
| Naa50 | 1.35003639 | 0.529525 | 1.345958335 | 0.000386 |
| Cycs | 1.693879438 | 0.670133 | 1.334981973 | 0.000387 |
| Set | 1.974986211 | 0.786808 | 1.325734271 | 0.000384 |
| Serbp1 | 2.831577665 | 1.142817 | 1.30842486 | 0.000319 |
| Tomm20 | 1.103917155 | 0.445758 | 1.302793847 | 0.000791 |
| Nhp2l1 | 1.220944438 | 0.508583 | 1.258974693 | 0.0011 |
| Ran | 3.943940088 | 1.6514 | 1.256347999 | 0.000559 |
| Actg1 | 3.746079527 | 1.591567 | 1.235259141 | 0.000887 |
| Ranbp1 | 3.15008491 | 1.34625 | 1.226364307 | 0.000961 |
| Tm4sf1 | 1.433282602 | 0.622267 | 1.200582617 | 0.003951 |
| Hnrnpab | 1.410359732 | 0.613292 | 1.198201036 | 0.001995 |
| Emg1 | 1.046006747 | 0.454733 | 1.196572139 | 0.003052 |
| Eif3j1 | 1.056864949 | 0.463708 | 1.183439563 | 0.003335 |
| Npm1 | 8.158128747 | 3.60795 | 1.178646427 | 0.001007 |
| Hspa9 | 1.252312576 | 0.553458 | 1.174229877 | 0.004018 |
| Anp32b | 1.598568558 | 0.718 | 1.152384473 | 0.002719 |
| Pdpn | 3.857074476 | 1.750125 | 1.140606118 | 0.005325 |
| Cxcl1 | 3.27193806 | 1.495834 | 1.129415288 | 0.019691 |
| Snrpd1 | 1.220944438 | 0.562433 | 1.114584784 | 0.005704 |
| Cnbp | 1.236628507 | 0.5744 | 1.102766727 | 0.005904 |
| Mif | 4.364997014 | 2.133059 | 1.034003877 | 0.007964 |
| Eif5a | 3.613368175 | 1.771067 | 1.029342363 | 0.007412 |
| Metap2 | 1.123220625 | 0.553458 | 1.017436082 | 0.013799 |
| Hspe1 | 3.553044833 | 1.786025 | 0.99294879 | 0.011249 |
| Ptges3 | 1.646827232 | 0.84365 | 0.963492274 | 0.019356 |
| Eif2s2 | 2.39121727 | 1.229575 | 0.959377184 | 0.017188 |
| Hspa8 | 6.341189692 | 3.317759 | 0.936088807 | 0.016356 |
| Ccl7 | 6.161426133 | 3.225017 | 0.935472409 | 0.027091 |
| Eif4a1 | 3.420333481 | 1.800984 | 0.926036374 | 0.021283 |
| Eif5 | 1.048419681 | 0.550467 | 0.925898879 | 0.034901 |
| Erh | 2.6083813 | 1.38215 | 0.916358316 | 0.024754 |
| Snrpd3 | 1.435695535 | 0.762875 | 0.910366447 | 0.034115 |
| Hnrnpc | 1.008606275 | 0.544483 | 0.885792723 | 0.046624 |
| Nap1l1 | 2.254886517 | 1.2206 | 0.885274271 | 0.036261 |
| Snrpe | 1.976192678 | 1.079992 | 0.871163001 | 0.040524 |
| Cxcl13 | 0.056704 | 1.103925 | -4.254010211 | 1.15E-08 |
| Mgp | 0.487413 | 8.310851 | -4.086163272 | 2.53E-14 |
| Angptl7 | 0.301617 | 3.425459 | -3.498439026 | 1.42E-10 |
| Apod | 0.220783 | 2.324525 | -3.38765564 | 2.85E-07 |
| Serpina3n | 0.159254 | 1.1847 | -2.885304013 | 1.36E-15 |
| Ogn | 0.256977 | 1.750125 | -2.760881119 | 8.42E-17 |
| C3 | 0.246119 | 1.433009 | -2.535001747 | 7.33E-12 |
| Tnxb | 0.225609 | 1.247525 | -2.460361531 | 1.21E-14 |
| Igfbp6 | 0.516368 | 2.773275 | -2.420738409 | 1.56E-15 |
| Igfbp4 | 0.32454 | 1.567634 | -2.266949267 | 9.73E-13 |
| Thbs4 | 1.264377 | 5.980342 | -2.238576573 | 4.21E-08 |
| Timp2 | 0.339017 | 1.534725 | -2.173664448 | 4.50E-13 |
| Serping1 | 0.483793 | 2.154 | -2.150395009 | 9.73E-13 |
| Col1a2 | 1.685434 | 7.386426 | -2.128737932 | 2.69E-13 |
| Gas1 | 0.330572 | 1.438992 | -2.117192779 | 1.74E-09 |
| Col1a1 | 0.733532 | 2.785242 | -1.921478978 | 5.10E-10 |
| Mgst1 | 0.494651 | 1.833892 | -1.886691513 | 3.36E-10 |
| Dpt | 0.297997 | 1.056058 | -1.821000491 | 2.45E-08 |
| Col3a1 | 3.463766 | 12.21198 | -1.815165069 | 4.44E-10 |
| Fn1 | 0.914502 | 3.21305 | -1.80975612 | 1.18E-07 |
| Gsn | 4.786054 | 16.63367 | -1.794523562 | 8.43E-10 |
| Col6a1 | 0.305236 | 1.032125 | -1.753534792 | 6.27E-08 |
| Cst3 | 1.610633 | 5.382009 | -1.73766815 | 5.39E-08 |
| Ly6c1 | 1.200435 | 3.748559 | -1.639910952 | 7.24E-08 |
| Ifi205 | 0.462077 | 1.3283 | -1.520290276 | 7.30E-06 |
| Pcolce | 0.735945 | 2.085192 | -1.499645907 | 1.13E-06 |
| S100a4 | 0.962761 | 2.665575 | -1.466439458 | 1.61E-05 |
| Plpp3 | 0.781791 | 2.151009 | -1.457369679 | 4.37E-06 |
| Plac8 | 0.437947 | 1.1847 | -1.432791808 | 0.000174152 |
| Gpx3 | 0.470522 | 1.271458 | -1.431276074 | 0.000632008 |
| Serpinf1 | 0.877101 | 2.366409 | -1.429153231 | 5.12E-06 |
| Lox | 0.907263 | 2.408292 | -1.405721225 | 6.74E-05 |
| Tgfbr2 | 0.413818 | 1.082983 | -1.385152234 | 3.80E-05 |
| Ly6a | 3.853455 | 9.854551 | -1.352053785 | 7.26E-06 |
| Dcn | 5.599213 | 14.29718 | -1.349855347 | 1.30E-05 |
| Tsc22d3 | 0.726293 | 1.803975 | -1.309981798 | 0.000171013 |
| Zbtb20 | 0.803507 | 1.95655 | -1.281399396 | 6.12E-05 |
| Rarres2 | 1.008606 | 2.426242 | -1.263843356 | 0.0003531 |
| Itm2b | 1.235422 | 2.92585 | -1.241348106 | 7.37E-05 |
| Serpine2 | 0.693718 | 1.624475 | -1.225129091 | 0.000994997 |
| Anxa1 | 1.02791 | 2.402309 | -1.222241011 | 0.000133339 |
| Adamts5 | 0.669589 | 1.549684 | -1.208240907 | 0.000427438 |
| S100a13 | 0.776965 | 1.750125 | -1.169192755 | 0.000282529 |
| Ifi27l2a | 0.834875 | 1.860817 | -1.153968062 | 0.000763429 |
| Tgfbr3 | 0.602027 | 1.313342 | -1.12316668 | 0.000836726 |
| Selm | 0.486206 | 1.035117 | -1.088171621 | 0.001879165 |
| Cd47 | 0.616505 | 1.298383 | -1.072462842 | 0.001581356 |
| Oat | 0.539291 | 1.1129 | -1.043268149 | 0.002598559 |
| Col4a1 | 0.68648 | 1.409075 | -1.035416191 | 0.004163722 |
| Fos | 2.158369 | 4.427667 | -1.034203304 | 0.005325169 |
| Lrp1 | 0.546529 | 1.109908 | -1.020201575 | 0.0041505 |
| Dusp1 | 1.087027 | 2.198875 | -1.014169584 | 0.003922084 |
| Col6a3 | 0.836082 | 1.6873 | -1.010906456 | 0.003586154 |
| Jund | 0.691305 | 1.373175 | -0.988174375 | 0.006420918 |
| Sparc | 3.808816 | 7.449251 | -0.965305101 | 0.00393932 |
| Lum | 0.62133 | 1.208633 | -0.958142093 | 0.017324861 |
| Nid1 | 0.721467 | 1.388134 | -0.942265964 | 0.009099872 |
| Nupr1 | 0.530845 | 1.020158 | -0.94080892 | 0.017321454 |
| Crip1 | 4.91032 | 9.366909 | -0.929292242 | 0.010905582 |
| Id3 | 0.902437 | 1.717217 | -0.926187488 | 0.018356925 |
| Fstl1 | 1.350036 | 2.554884 | -0.918088844 | 0.007828979 |
| Lgals3 | 0.920534 | 1.726192 | -0.905088106 | 0.014106911 |
| Lamp2 | 0.760074 | 1.391125 | -0.870281201 | 0.018359251 |
| Cd81 | 0.868656 | 1.582592 | -0.863584351 | 0.018001232 |
| Aebp1 | 0.770932 | 1.403092 | -0.862180103 | 0.019998474 |
| Cd9 | 1.259551 | 2.291617 | -0.861386792 | 0.021283225 |
| Igfbp7 | 0.969999 | 1.738159 | -0.839621621 | 0.032097152 |
| Malat1 | 59.85765 | 105.0524 | -0.808943417 | 0.022584899 |
| Laptm4a | 0.949489 | 1.657384 | -0.801883393 | 0.031361894 |
| Ifitm2 | 2.844849 | 4.927276 | -0.790132075 | 0.032602632 |
| Ebf1 | 1.380198 | 2.384359 | -0.786704304 | 0.039272538 |

**Table S2. Concentration of cytokines** **in culture medium of sorted FAPs.**

| Cytokine | Mean concentration in CTX 0 day FAP (pg/ml, n=3) | Mean concentration in CTX 1 day FAP (pg/ml, n=3) | P-Value |
| --- | --- | --- | --- |
| IFN-gamma | 0.21 | 0.81 | 2.35E-07 |
| IL-12p70 | 0.61 | 3.14 | 1.4E-09 |
| IL-13 | 0.98 | 2.38 | 1.83E-06 |
| IL-1beta | 0.56 | 1.88 | 7.38E-08 |
| IL-2 | 1.48 | 2.71 | 9.72E-05 |
| IL-4 | 0.73 | 0.86 | 8.96E-05 |
| IL-5 | 0.43 | 3.61 | 4.1E-06 |
| IL-6 | 104.52 | 11582.21 | 3.58E-06 |
| TNF-alpha | 4.86 | 33.63 | 2.45E-09 |
| GM-CSF | 4.08 | 62.79 | 5.17E-08 |
| IL-18 | 31.52 | 207.25 | 8.51E-08 |
| IL-10 | 1.89 | 3.46 | 0.004941 |
| IL-17A | 0.51 | 0.81 | 0.032104 |
| IL-22 | 5.35 | 12.29 | 0.004495 |
| IL-23 | 6.40 | 8.79 | 0.00048 |
| IL-27 | 1.06 | 1.30 | 0.020466 |
| IL-9 | 13.92 | 14.97 | 0.132953 |
| GRO-alpha (CXCL1) | 129.06 | 7354.33 | 0.001378 |
| IP-10 | 1.71 | 5.80 | 1.7E-05 |
| MCP-1 (CCL2) | 620.41 | 1892.47 | 1.43E-08 |
| MCP-3 (CCL7) | 308.20 | 365.33 | 0.000989 |
| MIP-1alpha | 0.58 | 1.46 | 1.46E-05 |
| MIP-1beta | 0.71 | 1.23 | 5.03E-08 |
| MIP-2 (CXCL2) | 4.69 | 361.74 | 2.03E-09 |
| RANTES | 1.85 | 7.19 | 3.21E-05 |
| Eotaxin (CCL11) | 180.88 | 270.73 | 3.03E-05 |
| IFN alpha | 9.21 | 8.43 | 0.312382 |
| IL-15/IL-15R | 0.44 | 0.85 | 0.009753 |
| IL-28 | 47.24 | 53.81 | 0.010085 |
| IL-3 | 0.24 | 0.30 | 0.000913 |
| G-CSF | 2.27 | 55.41 | 1.14E-07 |
| M-CSF | 0.76 | 0.81 | 0.071641 |
| LIF | 19.47 | 171.55 | 6.4E-09 |
| IL-1alpha | 2.16 | 4.18 | 0.009466 |
| ENA-78 (CXCL5) | 40.93 | 3321.56 | 7.58E-09 |
| IL-31 | 3.91 | 8.20 | 0.021689 |

Accessed by Mouse 36 cytokines multiplex kit. Red indicated the most abundant cytokines (>300pg/ml) secreted by sorted FAPs from muscles at 1 day after injury. P value was assessed by unpaired two-tailed Student’s *t*-test.

**Table S3. Antibodies list**

| **Antibodies** | **Isotype** | **Dilution** | **Source** | **Identifier** |
| --- | --- | --- | --- | --- |
| **IHC and IF antibodies** | | | | |
| WGA | Mouse monoclonal | 1:200 | Sigma | Cat#L4895 |
| Galectin-3/Mac-2 | Mouse monoclonal | 1:400 | Santa Cruz | Cat#sc-32790 |
| MyoD | Rabbit polyclonal | 1:200 | Santa Cruz | Cat#sc-304 |
| CD140A/PDGFRa | Rat monoclonal | 1:100 | BD | Cat#562776 |
| GROa/CXCL1 | Rabbit polyclonal | 1:50 | Abcam | Cat# ab17882 |
| **Flow cytometry antibody** | | | | |
| CD45 Percp-Cy5.5 | Rat anti mouse | 1:100 | BD | Cat#550994 |
| CD11b APC-Cy7 | Rat anti mouse | 1:100 | BD | Cat#557657 |
| F4/80 PE | Rat anti mouse | 1:100 | eBioscience | Cat#12-4801-83 |
| F4/80 APC | Rat anti mouse | 1:100 | eBioscience | Cat#17-4801-82 |
| Gr1 FITC | Rat anti mouse | 1:100 | Biolegend | Cat#108406 |
| CD3e PE-CF594 | Rat anti mouse | 1:100 | BD | Cat#562286 |
| CD45 FITC | Rat anti mouse | 1:100 | BD | Cat#553079 |
| Ly6A/E(Sca-1) PE-Cy7 | Rat anti mouse | 1:100 | BD | Cat#558162 |
| CD31 FITC | Rat anti mouse | 1:100 | BD | Cat#553372 |
| α7-integrin APC | Mouse monoclonal | 1:10 | R＆D | Cat#FAB3518A |
| IL1RL1 PE | Rat anti mouse | 1:100 | BD | Cat#566311 |
| CXCR2 APC | Mouse monoclonal | 1:10 | R＆D | Cat#FAB2164A |
| CCR2 PE | Mouse monoclonal | 1:10 | R＆D | Cat#FAB5538P |
| **Western Blot antibodies** | | | | |
| p-p44/42MAPK (ERK1/2) (Thr202/Tyr204) | Rabbit monoclonal | 1:1000 | Cell Signaling Technology | Cat#4370 |
| p44/42MAPK (ERK1/2) | Rabbit monoclonal | 1:1000 | Cell Signaling Technology | Cat#4695 |
| p-MAPKp38 (Thr180/Tyr182) | Rabbit monoclonal | 1:1000 | Cell Signaling Technology | Cat#4511 |
| MAPKp38 | Rabbit monoclonal | 1:1000 | Cell Signaling Technology | Cat# 8690 |
| p-SAPK/JNK (Thr183/Tyr185) | Rabbit polyclonal | 1:1000 | Cell Signaling Technology | Cat# 4668 |
| SAPK/JNK | Rabbit polyclonal | 1:1000 | Cell Signaling Technology | Cat#9252 |
| p-NF-κBp65 (Ser536) | Rabbit monoclonal | 1:1000 | Cell Signaling Technology | Cat#3033 |
| NF-κBp65 | Rabbit polyclonal | 1:1000 | Cell Signaling Technology | Cat#8242 |
| GAPDH | Mouse monoclonal | 1:2000 | Cell Signaling Technology | Cat#2118 |

**Table S4. Oligonucleotide sequences for realtime-PCR**

| Gene | Forward 5’­3’ | Reward 5’­3’ |
| --- | --- | --- |
| *Il1rl1* | TGTATTTGACAGTTACGGAGGGC | ACTTCAGACGATCTCTTGAGACA |
| *Il33* | TCCAACTCCAAGATTTCCCCG | CATGCAGTAGACATGGCAGAA |
| *Cxcl1* | CTGGGATTCACCTCAAGAACATC | CAGGGTCAAGGCAAGCCTC |
| *Cxcl5* | GTTCCATCTCGCCATTCATGC | GCGGCTATGACTGAGGAAGG |
| *Ccl2* | CGGCTGGAGCATCCACGTGTT | GTAGCAGCAGGTGAGTGGGGC |
| *Ccl7* | CCACATGCTGCTATGTCAAGA | ACACCGACTACTGGTGATCCT |
| *CD11b* | CCATGACCTTCCAAGAGAATGC | ACCGGCTTGTGCTGTAGTC |
| *Il6* | GGTGACAACCACGGCCTTCCC | AAGCCTCCGACTTGTGAAGTGGT |
| *Fn1* | ACGGTTTCCCATTACGCCAT | TGAGCTTAAAGCCAGCGTCA |
| *Myod1* | AGCATAGTGGAGCGCATCTC | GGTCTGGGTTCCCTGTTCTG |
| *Myog* | CAGCCCAGCGAGGGAATTTA | AGAAGCTCCTGAGTTTGCCC |
| *Ccna2* | TTGAACTACAAGACCAGCAGCC | GAGGAGCGTAGAGCCCAGGA |
| *Ccnd1* | CTGACAACTCTATCCGGCCC | TCATCCGCCTCTGGCATTTT |
| *Ccne1* | CTGTTTGGCCAATGGCTTTGA | TGAGTTATGTGTACTACTGTGACAA |
| *Cdk1* | AAGTGTGGCCAGAAGTCGAG | TCGTCCAGGTTCTTGACGTG |
| *Col1a1* | CGATGGATTCCCGTTCGAGT | CATTAGGCGCAGGAAGGTCA |
| *Col3a1* | AAGGCTGCAAGATGGATGCT | GTGCTTACGTGGGACAGTCA |
| *Actb* | GCAAGCAGGAGTACGATGAGT | AACGCAGCTCAGTAACAGTC |
